# Gut microbiota as an antioxidant system in centenarians associated with high antioxidant activities of gut-resident *Lactobacillus*

**DOI:** 10.1038/s41522-022-00366-0

**Published:** 2022-12-24

**Authors:** Lei Wu, Xinqiang Xie, Ying Li, Tingting Liang, Haojie Zhong, Lingshuang Yang, Yu Xi, Jumei Zhang, Yu Ding, Qingping Wu

**Affiliations:** 1 Guangdong Provincial Key Laboratory of Microbial Safety and Health, State Key Laboratory of Applied Microbiology Southern China, Institute of Microbiology, Guangdong Academy of Sciences, Guangzhou, Guangdong China; 2 The First Affiliated Hospital of Guangdong Pharmaceutical University, Guangzhou, Guangdong China; 3 School of Biology and Biological Engineering, South China University of Technology, Guangzhou, Guangdong China; 4 Department of Food Science and Technology, Institute of Food Safety and Nutrition, Jinan University, Guangzhou, Guangdong China

**Keywords:** Metagenomics, Symbiosis

## Abstract

The gut microbiota plays an important role in human health and longevity, and the gut microbiota of centenarians shows unique characteristics. Nowadays, most microbial research on longevity is usually limited to the bioinformatics level, lacking validating information on culturing functional microorganisms. Here, we combined metagenomic sequencing and large-scale in vitro culture to reveal the unique gut microbial structure of the world’s longevity town—Jiaoling, China, centenarians and people of different ages. Functional strains were isolated and screened in vitro, and the possible relationship between gut microbes and longevity was explored and validated in vivo. 247 healthy Cantonese natives of different ages participated in the study, including 18 centenarians. Compared with young adults, the gut microbiota of centenarians exhibits higher microbial diversity, xenobiotics biodegradation and metabolism, oxidoreductases, and multiple species (the potential probiotics *Lactobacillus*, *Akkermansia*, the methanogenic *Methanobrevibacter*, gut butyrate-producing members *Roseburia*, and SCFA-producing species uncl *Clostridiales*, uncl *Ruminococcaceae*) known to be beneficial to host metabolism. These species are constantly changing with age. We also isolated 2055 strains from these samples by large-scale in vitro culture, most of which were detected by metagenomics, with clear complementarity between the two approaches. We also screened an age-related gut-resident *Lactobacillus* with independent intellectual property rights, and its metabolite (L-ascorbic acid) and itself have good antioxidant effects. Our findings underscore the existence of age-related trajectories in the human gut microbiota, and that distinct gut microbiota and gut-resident as antioxidant systems may contribute to health and longevity.

## Introduction

Population aging is a growing problem facing the world today, and how to prolong life and maintain healthy aging is becoming a focus. Aging is a complex process, and scientists around the world are working hard to explore the underlying mechanisms of aging and try to delay aging by identifying key factors that may regulate aging^1,2^. Aging is related to a variety of factors, including genetics and the environment, with genetic factors accounting for 25–30% and environmental factors accounting for 70–75%^3,4^. Among various environmental factors, the gut microbiota is closely related to human health and longevity^5–8^, and thus, the gut microbiota emerges as a possible therapeutic target for aging and may provide new strategies for achieving healthy aging^9^.

Experiments in various animal models have shown that the gut microbiota plays an important role in the regulation of host longevity. Using a model organism - the African turquoise killifish (*Nothobranchius furzeri*), a naturally short-lived vertebrate, recolonizing the guts of middle-aged individuals with bacteria from young donors extends lifespan and delays behavioral decline, suggesting that the gut microbiota plays a key role in regulating vertebrate lifespan^10^. The Drosophila gut is the model of choice for studying human gut pathophysiology due to the simplicity of the microbiota and its physiological similarity to the mammalian gut. Studies have shown that microbially derived metabolites, when converted by host enzymes, can affect epithelial cell turnover and host longevity^11^. The naked mole rat (*Heterocephalus glaber*) is an excellent model for studying the biology of healthy aging and longevity. For the first time, scientists compared the gut microbial ecosystem of naked mole rats to that of humans and other mammals, and found some compositional features of the gut microbiome shared with the human gut microbial ecosystems of centenarians and Hadza hunter-gatherers. These data confirm the importance of the gut microbial ecosystem as an adaptive partner for mammalian biology and health^12^. In social insects like honeybees, the same genotype can show extreme differences in lifespan. Aging gut microbiota of short-lived (worker) bees produce *Proteobacteria* and deplete *Lactobacillus* and *Bifidobacterium*. In contrast, long-lived (queen) honeybees maintain youthful cellular function with much lower expression of oxidative stress genes, providing a unique perspective on age-related microbial succession^13^.

Clinical studies have also shown that the diversity and composition of the gut microbiota is non-linear with age. In centenarians, the abundances of *Roseburia* and *Escherichia* were significantly higher than in non-centenarians, while *Lactobacillus*, *Faecalibacterium*, *Parabacteroides*, *Butyricimonas*, *Coprococcus*, *Megamonas*, *Mitsuokella*, *Sutterella*, and *Akkermansia* were significantly lower in centenarians than in non-centenarians^14^. The age-related trajectories of the human gut microbiome are characterized by a loss of genes for short-chain fatty acid production and an overall decrease in glycolytic potential, while proteolytic functions are more abundant than in the gut metagenome of young adults^15^. A study using metagenomic sequencing to identify compositional and functional differences in the gut microbiota associated with age groups in Sardinia, Italy. The data showed that the gut microbiota of Sardinian centenarians was mainly characterized by depletion of *Faecalibacterium prausnitzii* and *Eubacterium rectale*, while enriched *Methanobrevibacter smithii* and *Bifidobacterium adolescentis* compared with young and old. Functional analysis showed that centenarians had higher metabolic capacity, especially glycolysis and fermentation of short-chain fatty acids (SCFAs), and lower genes encoding carbohydrate-degrading enzymes, including fiber and galactose^16^. Studies have shown that centenarians have a unique gut microbiome rich in microorganisms capable of producing unique secondary bile acids, including various isomers of lithocholic acid (LCA). These findings suggest that the metabolism of specific bile acids may be involved in reducing the risk of pathogenic infection and thus may contribute to the maintenance of intestinal homeostasis^17^. Although we believe that longevity appears to be achieved by maintaining gut microbiota homeostasis, whether changes in gut microbiota are a consequence or a cause of aging, and the exact relationship between gut microbiota and aging remains to be further explored.

Although numerous metagenomic studies have been conducted to explore the relationship between gut microbiota and lifespan, metagenomic analysis has not been able to provide viable microorganisms for further strain characterization or functional assessment^17,18^. The limitations of metagenomics make the actual gut microbiota of healthy, long-lived people far from fully understood. Recent studies have shown that culturomics seems to be able to fill this gap, as long as the optimal route and suitable culture conditions are found, all microorganisms seem to be able to be cultured^19^. This provides an effective complement to metagenomic sequencing to comprehensively characterize gut microbial composition. Currently, culturomics and metagenomics exhibit a high degree of complementarity, as only 15% of the tested species co-exist in both technologies^20,21^. However, these results were obtained by metagenomics and small-scale culture omics, and large-scale culture omics is still needed to prove this conclusion.

In this study, we combined metagenomic sequencing and large-scale in vitro culture to reveal the unique gut microbial structure of the world’s longevity town—Jiaoling, China, centenarians and people of different ages. Functional strains were isolated and screened in vitro, and the possible relationship between gut microbes and longevity was explored and validated in vivo, revealing associations of the gut microbiota with age and a number of clinical and metabolic parameters. We uncovered age-specific gut microbiota characteristics, including a core set of seven microbial taxa enriched in centenarians and the gut microbiota of centenarians exhibited higher xenobiotics biodegradation and metabolism, oxidoreductase. We revealed age-related gut microbial characteristics in all populations, including increased alpha diversity and increased levels of abundances of the health-related bacteria such as *Akkermansia*, *Lactobacillus*, and short-chain fatty acid (SCFA) producers, and targeted screening an age-related gut-resident *Lactobacillus* with independent intellectual property rights, which metabolites and itself have good antioxidant effects.

## Results

### Subject characteristics

**Fig. 1:**
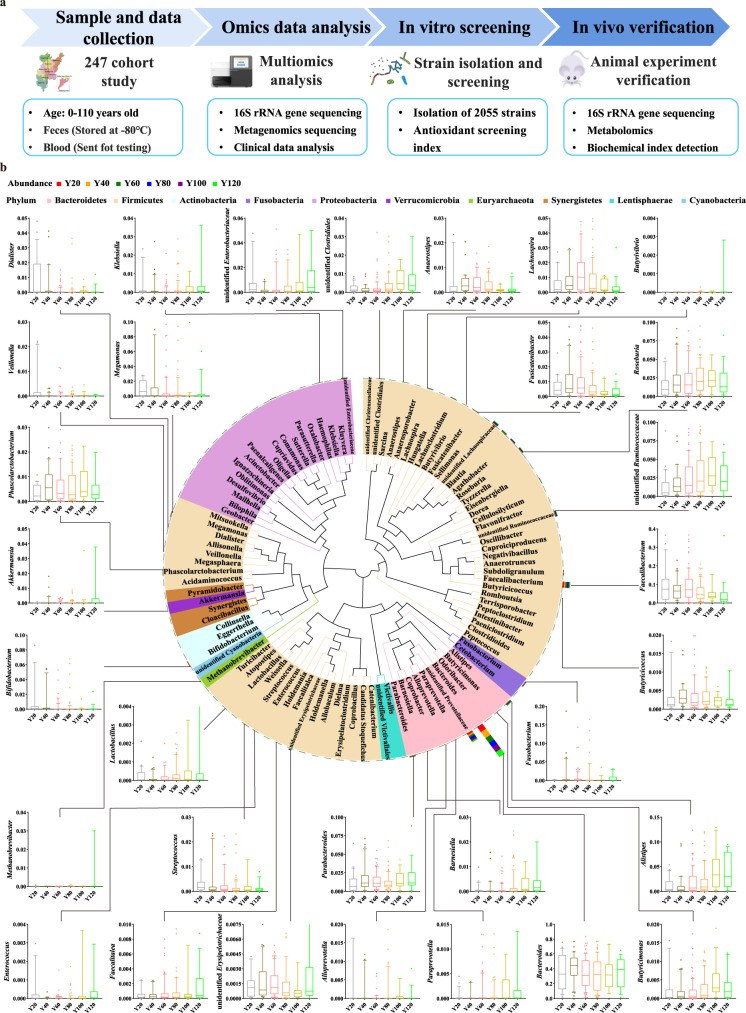
Overview of sampling regions and composition characteristics in gut microbiota. **a** Overview of sampling regions showing the 8 towns selected in Jiaoling (world’s longevity township), the Guangdong province of southern China. 16S rRNA gene and metagenomics sequence analysis and in in vitro and in vivo verification experiment process. **b** The phylogenetic relationship of top 100 species and age-related strains at the genus level. Statistical analysis between groups was expressed as mean ± SD, error bars were standard deviation.

A total of 247 participants from “World Longevity Township—Jiaoling, China” were recruited, including 18 centenarians (Y120 group), 19 persons aged 81–99 (Y100 group), 77 persons aged 61–80 (Y80 group), 81 persons aged 41–60 (Y60 group), 30 persons aged 21–40 (Y40 group), and 22 persons aged 0–20 (Y20 group) (Table 1). According to the multidisciplinary health assessment, the individuals were healthy with no apparent disease. All subjects were on an omnivorous diet and had not taken antibiotics in the 3 months prior to the study (Fig. 1a; Table S1).

**Table 1:** Demographical characteristics in world’s longevity townshipthe Jiaoling, China.

Group	Age (year)		Number	Male/Female	Weight (kg)	BMI (kg/m^2^)
	Segmentation	Mean ± SD				
Y20	0–20	9.9 ± 4.9	22	15/7	40.0 ± 21.3	18.7 ± 5.1
					(10.5-97.0)	(13.9-32.0)
Y40	21–40	32.6 ± 5.4	30	12/18	63.3 ± 10.4	23.6 ± 3.2
					(40.5-83.0)	(15.6-29.4)
Y60	41–60	52.2 ± 5.2	81	37/44	59.6 ± 9.6	23.2 ± 3.2
					(40.5-84.0)	(16.8-29.8)
Y80	61–80	68.2 ± 5.2	77	42/35	58.7 ± 10.6	23.1 ± 3.0
					(39.0-90.0)	(16.5-29.4)
Y100	81–99	87.2 ± 5.2	19	5/14	44.7 ± 8.7	19.7 ± 3.7
					(32.0-62.5)	(12.2-25.5)
Y120	100–110	101.8 ± 2.6	18	1/17	45.2 ± 12.5	20.6 ± 6.5
					(26.0-85.0)	(12.9-44.0)

### Age-related gut microbial characteristics

The Jiaoling cohort was established to study how gut microbiota are associated with aging. From this cohort, 16S rRNA gene amplicon and metagenomic data of fecal samples from all individuals aged 0–110 years were analyzed with host metabolic status, and isolated strains under the guidance of metagenomics, screened in vitro and verified the antioxidant function in vivo (Fig. 1a; Table S1).

Alpha-diversity indices, including observed species, Chao1, Shannon and ACE were markedly increased in older groups (Y80, Y100, Y120) than younger groups (Y20, Y40, Y60) (Fig. S1a-d). This is consistent with the results of Sepp et al.^22^, who showed that the richness and diversity of microbiota and the abundance of hereditary and environmental microbes were higher in people with longevity than young people. According to PCoA, the gut microbiota of the older groups (Y80, Y100, Y120) differed significantly from that of the younger groups (Y20, Y40, Y60) using the unweighted UniFrac distance (Fig. S1e). We did not observe significant differences between the Y20, Y40, Y60 groups or between the Y80, Y100, Y120 groups (Fig. S1a–d) for both alpha diversity and beta diversity. However, Bian et al.^23^ showed that gut microbiota of healthy aged Chinese is similar to that of the healthy young. Although microbial diversity has been a parameter to describe a healthy microbiome, it must be as a starting point for further inquiry of ecological mechanisms^24^.

The gut microbiota consisted of 270 genera and 107 families belonging to 22 phyla by 16S rRNA gene amplicon sequencing. According to the results of species annotations (Fig. S1f–h), the phylum levels of gut microbiota mainly included *Firmicutes*, *Bacteroidetes*, and *Proteobacteria* (Fig. S1f). *Methanobacteriaceae*, *Akkermansiaceae*, *Muribaculaceae*, *Ruminococcaceae*, *Christensenellaceae*, and *Lactobacillaceae* had a higher abundance in the Y120 centenarian group at the family level than in other groups. *Lachnospiraceae*, *Burkholderiaceae*, and *Bifidobacteriaceae* were less abundant (Fig. S1g). The potentially beneficial bacterial groups *Akkermansiaceae*, *Lactobacillaceae*, and *Christensenellaceae* were also found in the Y120 centenarian group. Their abundance gradually increased with age, which followed previous reports^25,26^. At the genus level, *Akkermansia*, *Methanobrevibacter*, *Klebsiella*, *Parabacteroides*, *Phascolarctobacterium*, *Barnosiella*, *Alisipes*, *Butyricimonas*, *Lachnoclostridium*, and *Blautia* species exhibited a higher abundance in the Y120 centenarian group than in other groups (Fig. S1h). This is consistent with the results of Palmas et al.^27^, who showed that the main biomarkers associated with centenarians belonged to the *Verrucomicrobia* phylum, including the *Akkermansia muciniphila* species, considered to be a significant biomarker of gut homeostasis for its ability to promote intestinal integrity. We analyzed the phylogenetic relationship of top 100 species at the genus level, and clearly observed that relative abundances of *Akkermansia*, *Methanobrevibacter*, *Klebsiella*, *Parabacteroides*, *Phascolarctobacterium*, *Barnosiella*, *Alisipes*, *Butyricimonas*, *Lachnoclostridium*, and *Blautia*, etc. gradually increases with age (Fig. 1b). Studies have shown that *Akkermansia* can prolong the lifespan of prematurely aging mice and enhance the intestinal barrier^28,29^.

**Fig. 2:**
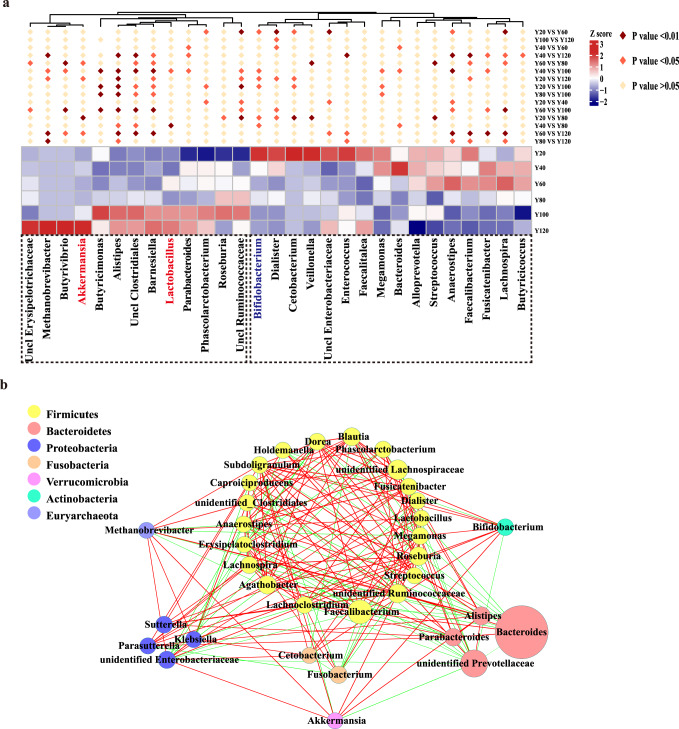
Age-related gut microbial characteristics. **a** Relative abundances of age-related gut microbiota *Lactobacillus*, *Akkermansia*, *Methanobrevibacter*, *Alistipes*, *Parabacteroides*, Uncl *Erysipelotrichaceae*, *Butyrivibrio*, *Butyricimonas*, *Barnesiella*, *Phascolarctobacterium*, *Roseburia*, Uncl *Clostridiales*, and Uncl *Ruminococcaceae* between younger (Y20, Y40, Y60) and older (Y80, Y100, Y120) groups by Wilcoxon rank-sum test, * indicates *p* < 0.05; ** indicates *p* < 0.01; *** indicates *p* < 0.001. **b** Co-occurrence network analysis of gut microbiota at the genus level. Different nodes represent different genera, the size of the node represents the average relative abundance of the genus, the nodes of the same phylum have the same color, and the thickness of the line between nodes is positively correlated with the absolute value of the correlation coefficient of species interaction. The positive and negative correspondence between the color of the connection and the correlation (red positive correlation, blue negative correlation).

**Fig. 3:**
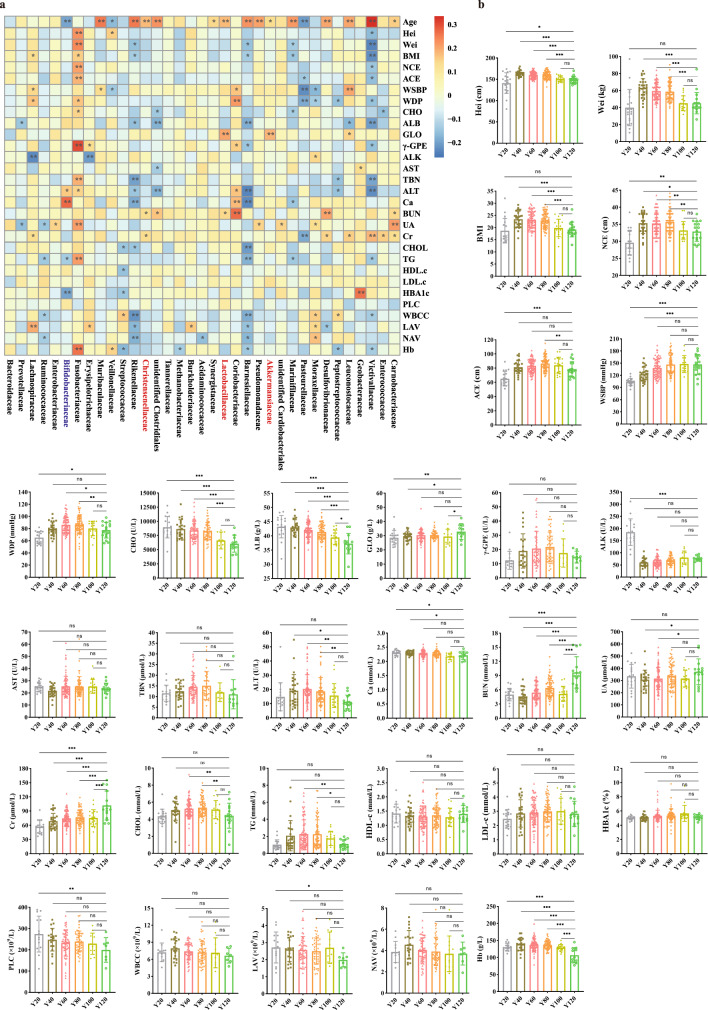
Correlation analysis of gut microbiota and clinical parameters. **a** Correlations between the relative abundance of selected bacterial family and clinical parameters. Correlations with *p* < 0.05 are shown. Spearman’s rho statistic adjusted using the Benjamini and Hochberg method. Age years old, Hei height, Wei weight, BMI body mass index, NCE neck circumference, ACE abdominal circumference, WSBP systolic blood pressure, WDP diastolic blood pressure, CHO cholinesterase, ALB albumin, GLO globulin, γ-GPE γ-glutamyl transpeptidase, ALK alkaline phosphatase, AST aspartate aminotransferase, TBN total bilirubin, ALT alanine aminotransferase, Ca calcium, BUN urea nitrogen, UA uric acid, Cr creatinine, CHOL total cholesterol, TG triglycerides, HDL.c high-density lipoprotein, LDL.c low-density lipoprotein. HBA1c glycated hemoglobin, PLC platelet count, WBCC white blood cell count, LAV absolute lymphocyte value, NAV absolute value of neutrophils, Hb hemoglobin concentration. **b** Clinical parameters of all the groups. Changes in clinical indicators of different age groups. * indicates *p* < 0.05; ** indicates *p* < 0.01; *** indicates *p* < 0.001; ns not significant, one-way ANOVA analysis of variance and expressed as mean ± SD, error bars were standard deviation.

Having well-characterized cohorts with diversity increase in gut microbiota, we next asked if we could identify age-associated human gut microbial characteristics among these cohorts. Of note, we found that high levels of 13 genera in the older groups (Y80, Y100, Y120). These genera included the potential probiotics *Lactobacillus*, *Akkermansia*, the methanogenic *Methanobrevibacter*, *Alistipes*, *Parabacteroides*, uncl *Erysipelotrichaceae*, *Butyrivibrio*, *Butyricimonas*, *Barnesiella*, *Phascolarctobacterium*, gut butyrate-producing members *Roseburia*, and SCFA-producing species such as uncl *Clostridiales*, uncl *Ruminococcaceae*. (Fig. 1b, Fig. 2a, Fig. S2). Thus, we defined these taxa as age-related core taxa with increased age, where were complex co-occurring network relationship (Fig. 2b). Intriguingly, we found that age-related *Barnesiellaceae* were significantly inversely associated with metabolic profiles (Fig. 3a), such as BMI, CHOL, and levels of TG; *Lactobacillaceae* were significantly correlated with clinical parameters, including GLO and BUN; *Akkermansiaceae* were significantly correlated with clinical parameters GLO (Fig. 3a). From the clinical parameters of each group, it can be observed that the body health index of the people in the longevity area was good (Fig. 3b).Of note, causal links between *Akkermansia* and host metabolic benefits have been demonstrated in mouse and human studies^30,31^, and a high abundance of *Alistipes* was reported to be associated with a low risk of atherosclerotic cardiovascular disease^32^, low TG levels or low BMI^33,34^. These data characterized the trajectory of gut microbiota with age.

### Age-related gut functional characteristics

**Fig. 4:**
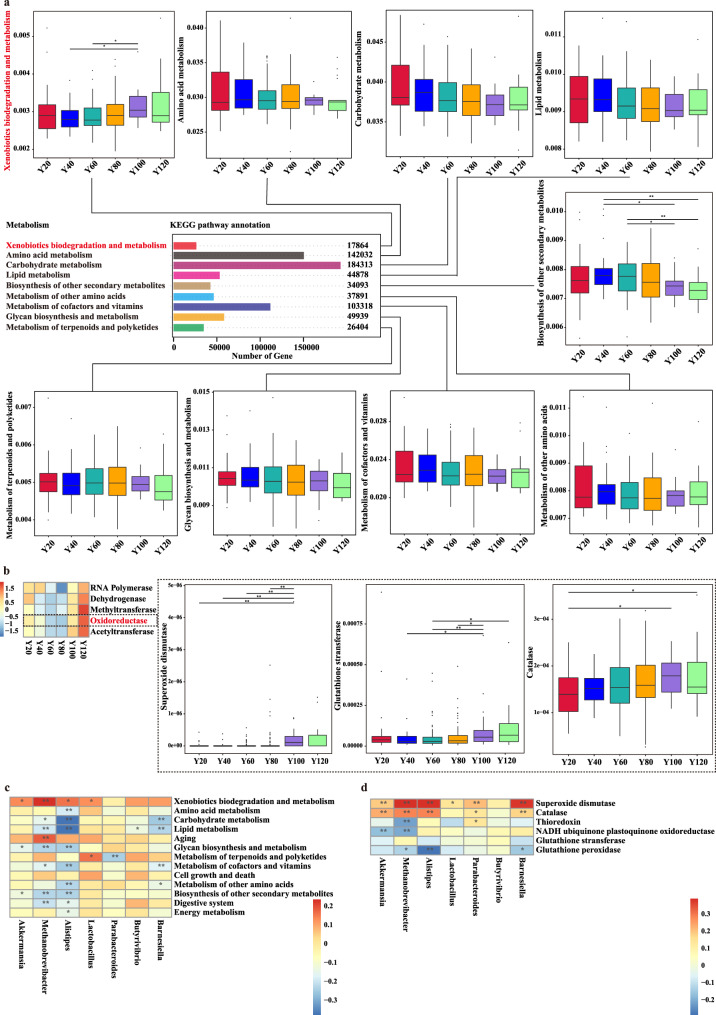
Age-related gut functional characteristics. **a** Box plot and bar chart showing the gut microbial KEGG pathway annotation (xenobiotics biodegradation and metabolism, amino acid metabolism, carbohydrate metabolism, lipid metabolism, biosynthesis of other secondary metabolites, metabolism of other amino acids, metabolism of cofactors and vitamins, glycan biosynthesis and metabolism, metabolism of terpenoids and polyketides). *P* values were obtained from two-sided Wilcoxon rank-sum tests. For all box and whisker plots, the center line represents median. The bounds of box represent the first and third quartiles. The upper whisker extends from the hinge to the largest value no further than 1.5 * interquartile range (IQR) from the hinge. The lower whisker extends from the hinge to the smallest value at most 1.5 * IQR of the hinge. **b** Heatmap showing the gut microbial functions of RNA polymerase, dehydrogenase, methyltransferase, oxidoreductase, acetyltransferase betwween six age groups. Box plot showing the changes of superoxide dismutase, glutathione stransferase, and catalase with age groups. **c**, **d** Correlations between the selected age-related bacterial genus and metabolic pathway and oxidoreductase. * indicates *p* < 0.05; ** indicates *p* < 0.01; *** indicates *p* < 0.001 by Wilcoxon rank-sum test.

At the functional level, we observed that a large number of metabolism pathway by metagenomic sequencing, such as carbohydrate metabolism, amino acid metabolism, metabolism of cofactors and vitamins, glycan biosynthesis and metabolism, lipid metabolism, metabolism of other amino acids, biosynthesis of other secondary metabolites, metabolism of terpenoids and polyketides, and xenobiotics biodegradation and metabolism. Compared to younger groups, the gut microbiota of older groups harbored higher abundances of a set of genes related to xenobiotics biodegradation and metabolism, which showing significant associations with increased age (Fig. 4a). Of note, we found that high levels of aminobenzoate degradation, atrazine degradation, benzoate degradation, caprolactam degradation, chlorocyclohexane and chlorobenzene degradation, dioxin degradation, ethylbenzene degradation, fluorobenzoate degradation, polycyclic aromatic hydrocarbon degradation, styrene degradation, toluene degradation, and xylene degradation significant associations with increased age (Fig. S3a).

We also observed that a large number of oxidoreductase, methyltransferase, acetyltransferase, dehydrogenase in Y120 group, where superoxide dismutase, glutathione stransferase, catalase significant associations with increased age (Fig. 4b, Fig. S3b). Together, our results show that age-related gut functional characteristics, such as xenobiotics biodegradation and metabolism, and oxidoreductase significant associations with increased age. Of note, age-related gut microbiota and function also exhibited significant associations, where *Akkermansia* significant associationswith xenobiotics biodegradation and metabolism, glycan biosynthesis and metabolism, biosynthesis of other secondary metabolites, superoxide dismutase, and catalase; *Lactobacillus* significant associationswith xenobiotics biodegradation and metabolism, metabolism of terpenoids and polyketides, and superoxide dismutase (Fig. 4c, d). These data indicated age-related gut functional characteristics.

### Targeted separation and screening of age-related strains

Having well-characterized age-related gut microbiota and function, we next asked if we could targeted separate, and screen these age-related gut microbiota and function strains. Indeed, we detected numerous significant associations between age and microbial diversity, the relative abundances of species or function in the Jiaoling, China (World Longevity Township).

**Fig. 5:**
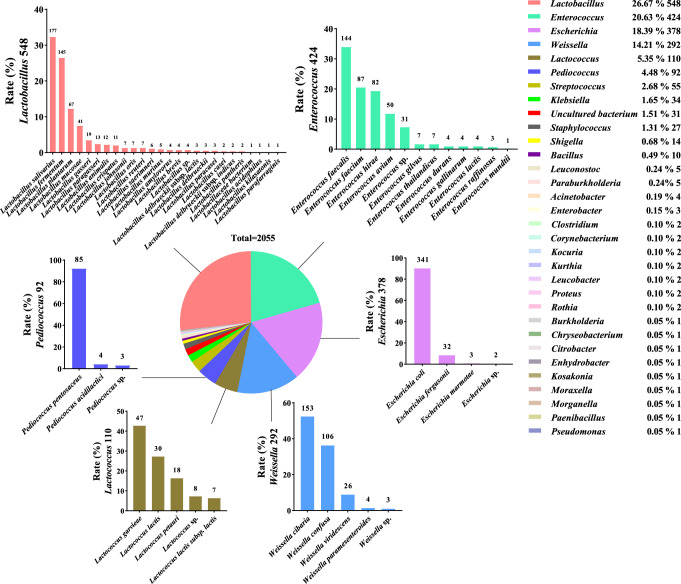
Targeted separation of age-related gut microbiota. The pie chart illustrating the proportion of all strains isolated from the fecal sample at the genus level, and a total of 2,055 strains were isolated. The bar graph showing the detailed composition of the top 6 genera isolated from the fecal sample, including *Lactobacillus, Enterococcus, Escherichia, Weissella, Lactococcus, Pediococcus*.

To verify the correlation between these age-related gut microbiota and function strains, we separated and purified 2055 strains, including *Lactobacillus* 26.67 %, *Enterococcus* 20.63 %, *Escherichia* 18.39 %, *Weissella* 14.21 %, *Lactococcus* 5.35 %, *Pediococcus* 4.48 %, *Streptococcus* 2.68 %, *Klebsiella* 1.65 %, Uncultured bacterium 1.51 %, *Staphylococcus* 1.31 %, *Shigella* 0.68 %, *Bacillus* 0.49 %, etc (Fig. 5, Fig. S4a). Since only a single medium was selected and cultured in an anaerobic environment, we did not isolate all the age-related strains analyzed by the above-mentioned big data. Fortunately, we have isolated a large number of *Lactobacillus*, mainly including *L. salivarius*, *L. fermentum*, *L. plantarum*, *L. mucosae*, *L. gasseri*, *L. paragasseri*, *L. animalis*, *L. crispatus*, etc (Fig. 5).

Next, some faecal isolation strains were selected for antioxidant probiotics screening. After preliminary screening (bacteria suspension) and re-screening (bacteria suspension, extracellular substance, intracellular substance, and bacteria fragment), a strain with strong antioxidant activity, named *Lactobacillus plantarum* 124 (LP124), was finally screened in vitro. The antioxidant screening indicators included scavenging rates of 1,1-diphenyl-2-picrylhydrazyl (DPPH), reducing activities of L-cysteine equivalent (μmol/L), scavenging rates of hydroxyl free radical (·OH), chelating rates of ferrous ion (Fe^2+^), scavenging rates of superoxide anion (O^2−^), and inhibition of lipid peroxidation (Table S2, 3, Fig. S5j). These results indicated that a strain with better antioxidant activity from gut microorganisms was isolated and screened in vitro and named it *Lactobacillus plantarum*124.

### Antioxidant mechanism of age-related gut-resident *Lactobacillus*

**Fig. 6:**
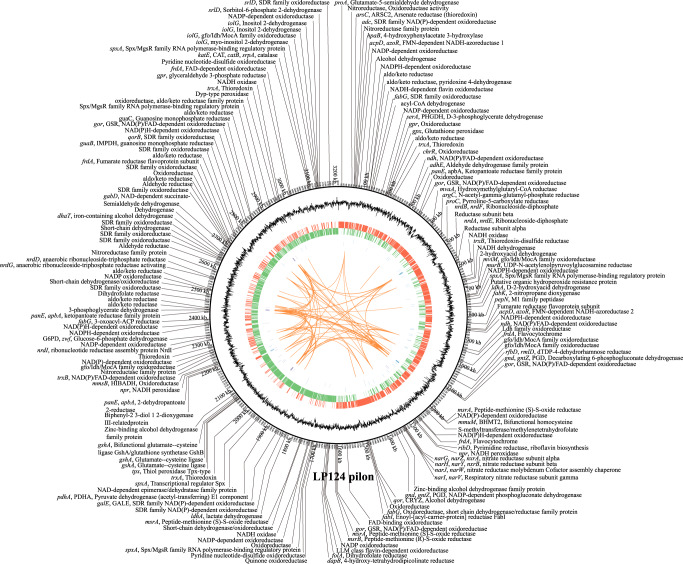
Whole-genome analysis of *Lactobacillus plantarum* 124. LP124 genome circle map and oxidoreductase related genes. The coding genes were annotated with National Center for Biotechnology Information (NCBI) NR database by BLAST. From the outside to the inside: the first circle is the genome location information, the second circle is the GC content information, the third circle is the coding gene on the positive chain (marked in red), and the fourth circle is the coding gene on the negative chain (marked in green), the fifth circle is the ncRNA information on the positive strand (marked in blue), the sixth circle is the ncRNA information on the negative strand (marked in purple), and the seventh circle is the information of long repetitive sequences in the genome (marked in orange), the eighth circle represents genes related to oxidoreductase.

Having well-known age-related strain LP124 with antioxidant activity in vitro, but whether there were oxidoreductase-related genes in vivo, we performed whole genome sequencing with total base length of sequence 3210286, (C+G)% 44.61%. Then the functions of genes were annotated by GO (Gene Ontology) database, and the pathways were annotated using KEGG (Kyoto Encyclopedia of Genes and Genomes) database. Intriguingly, we found that a large number of biological process, molecular function, and cellular component genes including metabolic process, cellular process, catalytic activity, binding, transporter activity, antioxidant activity. Finally, 12 antioxidant activity GO numbers and 140 antioxidant activity genes number were found (Fig. 6, Fig. S4b). The oxidoreductase-related gene clusters are annotated, mainly including *srlD*: SDR family oxidoreductase (EC:1.1.1.140, ko00051 Fructose and mannose metabolism), *ndh*: NAD(P)/FAD-dependent oxidoreductase (EC:1.6.99.3, ko00190 Oxidative phosphorylation), *gpx*: Glutathione peroxidase (EC:1.11.1.9, ko00480 Glutathione metabolism; ko00590 Arachidonic acid metabolism; ko04918 Thyroid hormone synthesis), *trxA*: Thioredoxin (ko04621 NOD-like receptor signaling pathway; ko05418 Fluid shear stress and atherosclerosis), *trxB*: Thioredoxin-disulfide reductase (EC:1.8.1.9, ko00240 Pyrimidine metabolism; ko00450 Selenocompound metabolism), *msrA*: Peptide-methionine (S)-S-oxide reductase (EC:1.8.4.11), and *katE, CAT, catB, srpA*: catalase (EC:1.11.1.6, ko00380 Tryptophan metabolism; ko00630 Glyoxylate and dicarboxylate metabolism; ko01200 Carbon metabolism; ko04011 MAPK signaling pathway-yeast; ko04016 MAPK signaling), etc (Fig. 6). We also found a large number of genes, such as carbohydrate metabolism, amino acid metabolism, energy metabolism, nucleotide metabolism, lipid metabolism, metabolism of other amino acids, and xenobiotics biodegradation and metabolism (Fig. S4c). Through pan-genomics analysis, we excavated the specific molecular target sequence 124_03212 of LP124 (Fig. S4d) and deposited it in the Culture Collection Center of the Institute of Microbiology, Guangdong Academy of Sciences, with the deposit number GDMCC61123 (Table S4). These results suggested that LP124 eprovided with a large of oxidoreductase-related genes in vivo.

**Fig. 7:**
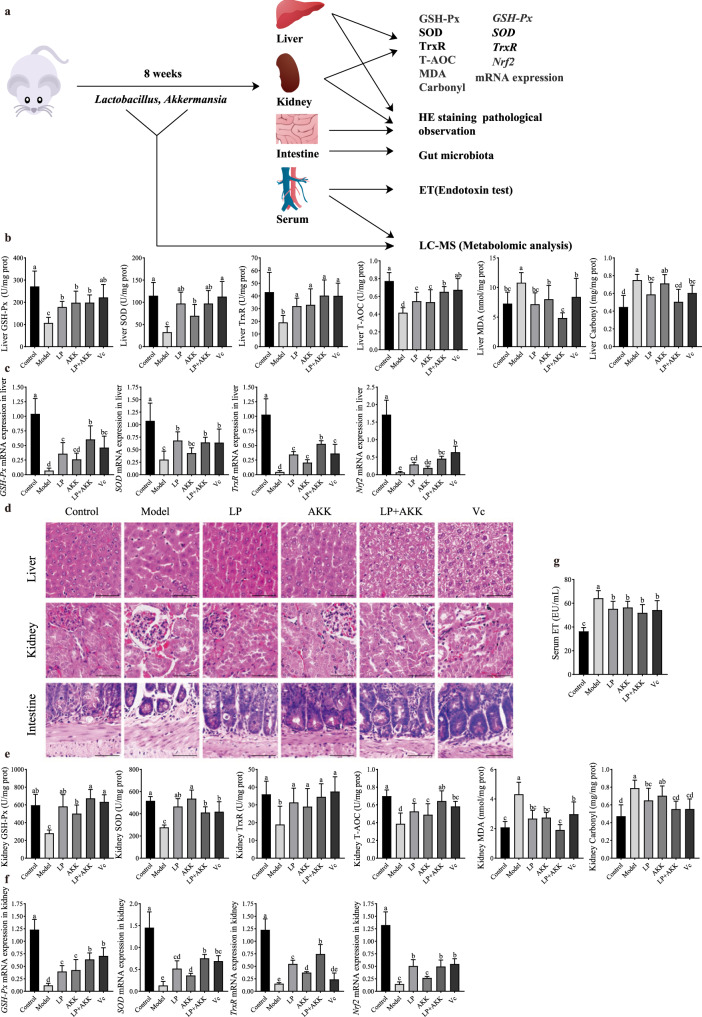
Effects of *Lactobacillus plantarum* 124 and *Akkermansia* on alleviating oxidative damage in mice. **a** Schematic of mice experimental design. **b**, **e** Mice liver and kidney antioxidant capacity determination, including glutathione peroxidase (GSH-Px) activity, superoxide dismutase (SOD) activity, thioredoxin reductase (TrxR) activity, total antioxidant (T-AOC) capacity, malondialdehyde (MDA) content, total carbonyl (Carbonyl) content. **c**, **f** The relative expression of *GSH-Px*, *SOD*, *Nrf2*, *TrxR* in liver and kidney tissues of oxidatively damaged mice. **d** Histomorphological analysis of liver kidney and intestine from different mice groups (original magnification, ×400; scale bar = 50 µm). **g** Mice serum endotoxin determination. Different letters (a, b, c, d) in the figure indicate significant differences between groups (*p* < 0.05, one-way ANOVA analysis), and the same letters indicate no significant differences. Statistical analysis between groups was expressed as mean ± SD, error bars were standard deviation.

To further test whether the gut-resident LP124 can also alleviate oxidative stress in animals, LP124 was intragastrically injected into mice (Fig. 7a, Fig. S5k,l). The gut microbial profiles of recipient mice were analysed by 16S rRNA gene amplicon sequencing at 8 weeks (Fig. S5a–c). As expected, LP124 could regulate the composition and function of the gut microbiota of oxidatively damaged mice. Additionally, at the genus level, the abundance increased in *Akkermansia*, *Bacteroides*, *Alloprevotella*, *Prevotellaceae*_UCG-001, *Lachnospiraceae*_NK4A136 compared to model group (Fig. S5a). Moreover, at the functional level, we observed that a large number of carbohydrate metabolism amino acid metabolism, lipid metabolism, xenobiotics biodegradation, and metabolism in *Lactobacillus*. These results revealed profound changes in the composition and function of the gut microbiota of the LP124 group, indicating the importance of LP124 in the development of gut microbes.

Glutathione peroxidase (GSH-Px), superoxide dismutase (SOD), thioredoxin reductase (TrxR), total antioxidant capacity levels (T-AOC), malondialdehyde (MDA), carbonyl, and endotoxin (ET) are thought to play key roles in the pathophysiology of oxidative stress damage. At 8 weeks, model mice exhibited significantly lower GSH-Px, SOD, TrxR, T-AOC, and higher MDA, carbonyl levels compared with control mice in the liver. Notably, LP mice were significantly higher GSH-Px, SOD, TrxR, T-AOC, and lower MDA, carbonyl levels compared with model mice (Fig. 7b). Similarly, the kidneys of LP mice also showed similar results to the liver (Fig. 7e). We next examined alterations in genes *GSH-Px*, *SOD*, *TrxR*, and nuclear factor-erythroid 2-related factor 2 (*Nrf2*) messenger expression in the liver and kidney. Particularly, the levels of *GSH-Px*, *SOD*, *TrxR*, and *Nrf2* mRNA were significantly increased in the LP group compared with those in the model group for mice (Fig. 7c, f). To determine how LP124 affect the liver, kidney, we examined the structure of them. Histomorphological analysis revealed a significant structural change of liver in model group compared with these in control group (Fig. 7d). In addition, differences in kidney morphology were evident in the model group compared with control group (Fig. 7d). The model mice exhibited some serious lesions in the liver and kidney. However, in the LP group, liver and kidney morphology resem-bled that in controls. Furthermore, no visible histopathological abnormalities were observed in the LP and vitamin C (Vc) groups (Fig. 7d). These results showed that LP124 has the function of relieving oxidative stress in the liver and kidneys.

Impaired body health responses in the intestine are known to be associated with gut barrier dysfunction^35^. We investigated if gut-resident LP124 affected gut barrier function. LP124 markedly decreased plasma endotoxin levels in LP mice (Fig. 7g). We next examined the effect of LP124 on intestinal histopathological changes. There was a significant structural change of intestine in model group compared with these in control group and no obvious difference in the morphology of the epithelium in LP mice compared with Vc group (Fig. 7d). These results demonstrated that LP124 has the function of protecting the intestinal barrier.

**Fig. 8:**
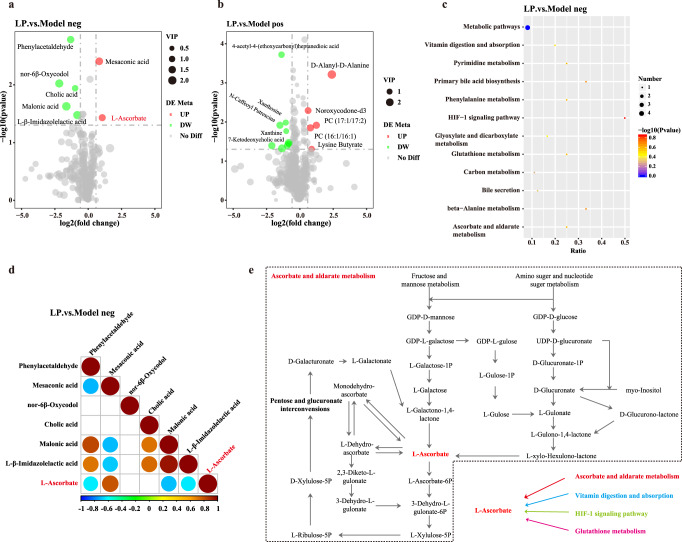
Analysis of serum metabolome of *Lactobacillus plantarum* 124 regulating oxidative damage in mice. **a**, **b** Features identified by ultra-high-resolution mass spectrometry of different metabolites significantly up-regulated and down-regulated in the LP group compared to the model group. The volcano chart can visually display the overall distribution of different metabolites. The significantly up-regulated metabolites are represented by red dots, the significantly down-regulated metabolites are represented by green dots, and the size of the dot represents the VIP value. **c** KEGG pathway enrichment bubble chart. The larger the value of abscissa, the higher the enrichment of differential metabolites in the pathway. The color of the dot represents the *p*-value of the hypergeometric test. The smaller the value, the greater the reliability of the test and the more statistically significant. The size of the dot represents the number of different metabolites in the corresponding pathway. The larger the dot, the more differential metabolites in the pathway. **d** Correlation diagram of different metabolites (the pos is the positive ion mode, the neg is the negative ion mode). Positive correlation is red, and negative correlation is blue. The part without color indicates *p*-value > 0.05, The figure shows the correlation of the top20 differential metabolites sorted by *p*-value from small to large. **e** Diagram of related pathways of L-ascorbate.

To determine the mechanism by which gut-resident LP124 exerts antioxidant activity at a distance, we characterized the small-molecule substance present in the serum of LP124-treated animals relative to model group by LC-MS. Subsequent tandem mass spectrometry identified candidate small molecules that were significantly enriched in the serum of LP124-treated animals, of which 2 were negatively identified as L-ascorbate and mesaconic acid (Fig. 8a, d, Fig. S5f, h), while 5 were positively identified as lysine butyrate, D-alanyl-D-alanine, noroxycodone-d3, PC (16:1/16:1) and PC (17:1/17:2) (Fig. 8b, Fig. S5d, g, i). Further analysis revealed that 15 metabolic pathways were involved in the above-mentioned metabolites with significant differences (Fig. 8c, Fig. S5e). Fortunately, among these 7 significantly enriched small molecules, we have detected a very important metabolite L-ascorbate, which is well known to have a very strong antioxidant effect^36–38^. To corroborate these findings, we determined the oxidative activity of Vc (L-ascorbate) in vivo. We observed a significantly higher levels in the liver and kidney of GSH-Px, SOD, TrxR, T-AOC, and lower MDA, carbonyl levels in Vc mice compared with model mice (Fig. 7b, e). Likewise, the expression levels in the liver and kidney of *GSH-Px*, *SOD*, *TrxR*, and *Nrf2* mRNA were significantly increased in the Vc group compared with those in the model group for mice (Fig. 7c, f). In addition, no obvious difference in the morphology of the epithelium in Vc mice compared with control group (Fig. 7d).

Finally, to determine whether the origin of L-ascorbate is produced by LP124, we performed metabolomics analysis on it (Table S5,6). Subsequent mass spectrometry showed an accumulation of L-ascorbate in LP124 culture supernatants (Table S5). Further analysis revealed that some metabolic pathways were involved in the L-ascorbate metabolites with ascorbate and aldarate metabolism, vitamin digestion and absorption, HIF-1 signaling pathway, glutathione metabolism. Especially, L-ascorbate as a metabolite of fructose and mannose metabolism, amino suger and nucleotide suger metabolism showed in Fig. 8e. Together, these data demonstrate that the gut-resident *Lactobacillus*-derived small molecule L-ascorbate can act as effective substance involved in the antioxidant response.

## Discussion

**Fig. 9:**
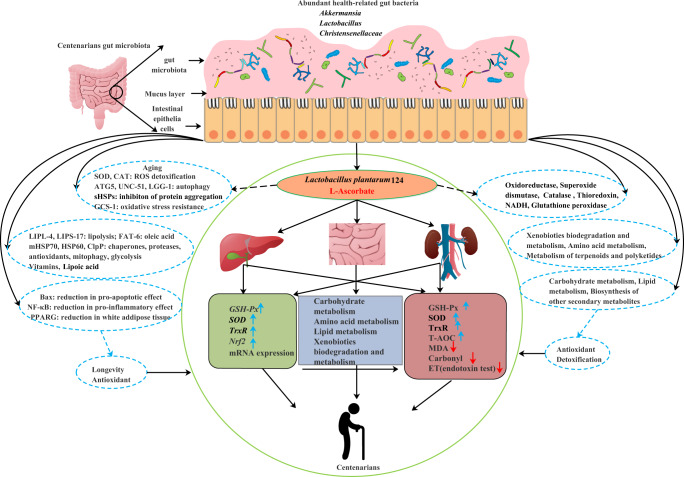
Structure and function characteristics of gut microbiota in centenarians and potential longevity mechanism. The existence of age-related trajectories in the human gut microbiota, and that distinct gut microbiota and gut-resident as antioxidant systems may contribute to health and longevity.

Jiaoling, China, longevity township in the world has a high incidence of centenarians, a relatively single population, and a unique lifestyle and diet. It is an ideal area for studying longevity. Therefore, an in-depth study of the gut microbiota of centenarians in Jiaoling, a town of longevity in the world, will help deepen our understanding of the mechanisms of longevity. Although the gut microbiota is considered to be an important determinant of human health^39^, the structure of the gut microbiota and the mechanisms that influence longevity in long-lived populations are not fully understood. Furthermore, how to thoroughly analyze the composition of the gut microbiota remains a great challenge. The main purpose of this study was to dig out the regional longevity factors belonging to the “World’s Longevity Township—Jiaoling, China” by characterizing the gut microbiota characteristics of people of different ages only in China. So we in-depth explored the characteristics of the gut microbiota of the longevity population in Jiaoling, the world’s longevity township, through metagenomics and culturomics without selecting the big data of other countries or even the world. To the best of our knowledge, this was the first study to combine metagenomic sequencing of fecal microbes with large-scale culture omics in Jiaoling, a town of longevity in the world, to thoroughly explore the relationship between gut microbiota and healthy longevity. In this study, we performed a comprehensive analysis of cohort in the world’s longevity town—Jiaoling, China, demonstrating the existence of age-related compositional and functional characteristics of gut microbes. We also demonstrate a previously underappreciated role of microbes in antioxidant responses. Furthermore, we identified *Lactobacilli* as mediators of this effect, in part because of their production of L-ascorbic acid, as we detected L-ascorbic acid in both the culture supernatant of in vitro cultures of *Lactobacillus* and in the serum of *Lactobacillus plantarum* 124 gavage animals (Fig. 9).

In our study, the elderly, especially the centenarian group, showed higher gut microbial diversity than the younger group, compared with the general youth living in the same region. The gut microbiome structure and function of centenarians have unique characteristics. However, studies have shown that centenarians are often characterized by reduced alpha diversity, decreased butyrate-producing bacteria (eg, *Faecalibacterium*, *Rosetella*, *Eubacterium*, and *Ruminococcus*), and increased opportunistic pathogens^40^. This may be due to longer colonic transit times in middle-aged people than in younger adults^41,42^, and longer colonic transit times are associated with higher gut microbial diversity^43^. In addition, our study also found that the elderly population also has a higher abundance of some healthy bacterial species, such as *Akkermansia*, *Lactobacillus* and many SCFA-producing bacteria (Fig. 9). *Akkermansiaceae*, *Lactobacillaceae* and *Barnesiellaceae* etc. were significantly correlated with clinical parameters. It is necessary for us to distinguish chronological aging from biological aging. Chronological age represents a person’s actual age and is calculated based on the time elapsed in a person’s life^44^. Biological age refers to an individual’s overall health status at a certain point in time of physiological age. Generally, the environment, diet, life, and psychological factors should be considered. Biological age has been revealed as a better predictor than chronological age, and its measurement can facilitate the assessment of colonoscopy-related colorectal adenoma risk^45^. Dyslipidemia, Diabetes, Inflammaging are also age-associated conditions. They could at least perform revalidate the age-associated taxonomic markers with these biomarkers. The extent of biological aging for individuals could be based on the age-associated residuals of these biomarkers and the taxonomic abundances of the above taxa could be associated with these age-adjusted residuals. Meanwhile, the abundance of *Methanobrevibacter* also gradually increased with age. Loss of *Akkermansia* in older adults and its roles in the maintenance of colonic mucus thickness and anti-inflammatory immune status have been reported in mice^46^. Consistent with previous findings, age-related significantly increased abundances of *Methanobrevibacter*, methanogenic genes, and microbial diversity may be associated with prolonged colonic transit time, which was observed in older adults^47,48^. *Methanobrevibacter* is gradually enriched with age, which may have a role in longevity^49^.

Our analyses revealed that older adults, especially the centenarians exhibited higher abundances of several health-promoting functions, including xenobiotics biodegradation and metabolism, and oxidoreductase significant associations with increased age. Centenarians are long-lived individuals so they may have an important history of exposure to external pressure. In addition, because of their reduced mobility, these people spend more time in their homes than do younger people, and thus incur increased exposure to indoor pollutants. Therefore, it is easy to speculate that their microbiome can better degrade these xenobiotics. This exposure and response could drive the accumulating xenobiotic-degrading ability in the gut microbiota of longevity people. Here, we speculate that the ability of long-lived people to degrade xenobiotics may be the result of a top-down selection process related to the lifestyle habits of these particularly long-lived individuals. Previous studies found that the gut microbiota of Italian adults can also degrade xenobiotics^50^, which may be a functional response to exposure to these compounds^51^.

Indeed, many systemic human diseases can be regulated by the collective microbial compositon within the gut^52^. This recognition has prompted attempts to modulate the microbiome therapeutically through the use of probiotics. However, the possibly diverse mechanisms by which specific bacteria and their products elicit their distinct beneficial effects remain unknown. Therefore, identification of molecular pathways affected by probiotics would allow for the more targeted use of probiotics or exploitation of microbial-derived small molecules. Using in vitro large-scale culture techniques, we isolated 2055 strains from fecal samples and performed targeted isolation and screening of one *Lactobacillus* strain, identifying L-ascorbic acid as a small molecule derived from LP124, though it is likely that many other small molecules in response to LP124 that we could not identify using our specific metabolomic platform. This suggests that culturomics is an important complement to metagenomics for a comprehensive understanding of the gut microbiota. Although the number of strains of gut microbiota species retrieved by culturomics does not reflect the actual abundance of each species, the higher segregation rates of culturomics still indicate, to some extent, the relatively higher relative abundance of this species in feces. Our study found that the number of *Lactobacillus* and *Enterococcus* strains cultured in the world’s longevity town - Jiaoling, China, is higher, suggesting that these two taxa may be more abundant in centenarians. These findings are consistent with previous literature reports on metagenomic data^53^. We found by culturomics that *L. salivarius* was the most abundant species of *Lactobacillus*. In order to ensure the accuracy of culturomics for *Lactobacillus* isolation, we analyzed the classification level of gut microbiota at the species level by metagenomics, and found that the top 20 species were *L. mucosae, L. salivarius, L. delbrueckii, L. fermentum, L. rogosae, L. johnsonii, L. amylovorus, L. reuteri, L. ruminis, L. equicursoris, L. murinus, L. gasseri, L. bifermentans, L. casei, L. ceti, L. plantarum, L. crispatus, L. iners, L. ingluviei, L. brevis*. Here we can clearly see that *L. salivarius* ranks second at the species level. This shows that the abundance of *L. salivarius* in the gut microbiota is very high, so it is normal that we can isolate a large number of *L. salivarius*. This result is also consistent with the results reported by Kristina et al. regarding the presence of large amounts of *Lactobacillus* in the colon^54^. As we know, the vast majority of strains in the gut microbiota are unculturable. Metagenomic analysis has not been able to provide viable microorganisms for further strain characterization or functional assessment. Culturomics seems to be able to fill this gap. This provides an effective complement to metagenomic sequencing to comprehensively characterize gut microbial composition. Currently, culturomics and metagenomics exhibit a high degree of complementarity and a certain degree of mutual validation. Therefore, culturomics contributes to further understanding of gut microbiota composition and highlights microbial dark matter. Combining culturomics and metagenomic data provides insight into the structure and function of gut bacterial communities. Future studies examining transcriptomic, proteomic, and lipidomic studies in animals exposed to a range of functional strains will contribute to our comprehensive understanding of the causal relationship between gut microbiota and human health and longevity.

In conclusion, this study increases our understanding of the relationship between gut microbiota and human health and longevity. Through the combination of metagenomics and in vitro large-scale culture, we have demonstrated for the first time the structural and functional characteristics of the gut microbiota of a healthy and long-lived population in Jiaoling, the world’s longevity township, and its unique enriched gut microbes. Furthermore, we establish *Lactobacillus* as a powerful driver of human health and add significant literature support for its potential as an antioxidant. These data also suggest that the gut microbiota, in addition to its intrinsically encoded specific biocatalytic activity, can induce host bioprocessing pathways with potential effects on ingested foreign organisms. To elucidate age-related trajectories of the gut microbiota, as well as generational-related differences that cannot be captured in cross-sectional data, further controlled longitudinal human studies across a broad age range are needed.

## Methods

### Study cohort

The overall research objectives of the Jiaoling (world’s longevity township, Jiaoling County, Meizhou City, Guangdong Province, China) cohort are to study how the gut microbiota changes in relation to age and how this may affect health. The study was approved by the ethics committee of The First Affiliated Hospital/School of Clinical Medicine of Guangdong Pharmaceutical University. The First Affiliated Hospital/School of Clinical Medicine of Guangdong Pharmaceutical University: 2021(13).

Based on the national civil registration system, a total of 247 participants were randomly drawn from eight towns. Participants were eligible based on the following criteria: (1) born in Jiaoling; (2) 5 years or longer continuous residence in Jiaoling backtracking from the sampling point; (3) aged 0–110 years. All enrolled participants signed an informed consent form before their physical examination and biomaterial collection. Subsequently, 247 participants meeting the following additional criteria were selected for the metagenomic study: (1) with fecal and blood samples; (2) without antibiotic treatment in the past month before biomaterial collections; (3) without severe diseases (end-stage cancer, renal or liver disease). Fecal samples were freshly collected from each subject and immediately frozen at −20 °C, transported to the laboratory on an ice pack, and stored in a −80 °C refrigerator until analysis. For age-related group comparison analyses, we divided subjects from the Jiaoling cohort into six age groups using a cutoff of 20 years. Clinical measurements, height, weight, and waist circumferences were measured by trained staff according to standardized protocols. The derived anthropometric parameters including BMI (kg/m^2^) were calculated and stored for further analyses. Blood pressure (mm Hg) was measured three times using an automatic manometer, and the mean of the measures was used in the analysis. Statistics of the factors are summarized in Table 1, Table S1, and Fig. 3.

### 16S rRNA gene amplicon and metagenomic sequencing analysis

Microbial DNA was extracted using the QIAamp DNA stool mini kit (QIAGEN, Hilden, Germany) with additional bead-beating and heating steps using standard methods^55^. Using Cutadapt (v1.9.1, http://cutadapt.readthedocs.io/en/stable/)^56^ to cut and filter reads, an average of 85,170 reads were measured per sample; after quality control (https://github.com/torognes/vsearch/)^57^, an average of 80,024 clean reads^58^ were obtained and the quality control efficiency reached 94.01%. Uparse software (v7.0.1001, http://www.drive5.com/uparse/)^59^ was used to cluster sequences into operational taxonomic units (OTUs) and 3,436 OTUs were obtained. Subsequently, we used the Mothur method and the SSUrRNA^60^, database of SILVA132 (http://www.arb-silva.de/)^61^, for species annotation analysis of the OTUs (threshold set to 0.8–1). QIIME software (v1.9.1) was used to calculate the Shannon index of community diversity. Theprincipal coordinates analysis (PCoA) diagrams were plotted using R software (v2.15.3) with the WGCNA, stats, and ggplot2 software packages. The differences among groups of alpha diversity and beta diversity index were analysed using R software. Linear discriminant analysis effect size (LEfSe) was performed to explain the differences between groups^62^. Graphviz-2.38.0 was used to draw the co-occurrence network graph. We used DIAMOND software^63,64^ to perform common functional database annotations on non-redundant gene sets (e-value ≤ 10^-5^). A total of 3,264,476 (60.85%) ORFs were compared to the KEGG database^65,66^, 3,204,116 (59.72%) ORFs were compared to the eggNOG database^67^, and 165,799 (3.09%) ORFs were compared to the CAZy database^68^.

### Targeted separation and screening of age-related strains

All samples were cultured on MRS (De Man, Rogosa, Sharpe) agar plates and incubated at 37 °C for 24 h with aerobic and anaerobic culture. Eight to ten suspicious colonies with typical morphology was selected from each sample for identification. We obtained 2055 isolates from fecal samples. After identifying isolates by MALDI-TOF MS (Biomerieux, France) or 16S molecular identification, we finally counted *Lactobacillus* 548, *Enterococcus* 424, *Weissella* 292, *Escherichia* 378, *Lactococcus* 110, *Pediococcus* 92, *Streptococcus* 55, *Klebsiella* 34, etc. 185 strains were randomly selected to be screened by antioxidant indicators included scavenging rates of 1,1-diphenyl-2-picrylhydrazyl (DPPH)^69^, reducing activities of L-cysteine equivalent (μmol/L)^70^, scavenging rates of hydroxyl free radical (·OH)^71^, chelating rates of ferrous ion (Fe^2+^)^72^, scavenging rates of superoxide anion (O^2−^)^73^, and inhibition of lipid peroxidation^74^ with preliminary screening (bacteria suspension), the same with re-screening (bacteria suspension, extracellular substance, intracellular substance, bacteria fragment).

Preparation of bacteria suspension, extracellular substance, intracellular substance, and bacteria fragment. The *Lactobacillis* obtained by self-isolation were inoculated into 1.5 mL of MRS liquid medium, cultivated at 37 °C for 24 h as the inoculum, and inoculated into 50 mL of MRS liquid medium with 2% inoculum, and cultured for 24 h. This obtains the culture solution of the strain. Centrifuge at 10,000 r/min for 10 min at 4 °C. The supernatant is the extracellular substance. Collect 10–15 mL in a 15 mL centrifuge tube; Resuspend the cells with sterile saline, adjust the cell density to 10^9^ CFU/mL, and OD_600_ = 1.0 ± 0.1, which is the bacteria suspension; take at least 3 mL to 15 mL centrifuge tubes, and sonicate them in ice bath (750 W, break for 5 s, intermittent for 5 s, working time 7.5 min, total time 15 min), centrifuge at 10,000 r/min for 25 min at 4 °C, collect the supernatant as intracellular substance; collect the precipitate, add the corresponding volume of normal saline, which is the bacteria fragment.

### Whole genome sequencing

Next generation sequencing library preparations were constructed following the manufacturer’s protocol. For each sample, 200 ng genomic DNA was randomly fragmented to <500 bp by sonication (Covaris S220). The fragments were treated with End Prep Enzyme Mix for end repairing, 5’ Phosphorylation and dA-tailing in one reaction, followed by a T-A ligation to add adaptors to both ends. Size selection of Adaptor-ligated DNA was then performed using Beads, and fragments of~470 bp (with the approximate insert size of 350 bp) were recovered. The PCR products were cleaned up using Beads, validated using an Qsep100 (Bioptic, Taiwan, China), and quantified by Qubit3.0 Fluorometer (Invitrogen, Carlsbad, CA, USA).

Then libraries with different indices were multiplexed and loaded on an Illumina HiSeq/Novaseq instrument according to manufacturer’s instructions (Illumina, San Diego, CA, USA) or a MGI2000 instrument according to manufacturer’s instructions (MGI, Shenzhen, China). For Pacbio, Genomic DNA was sheared, then 10Kb double-stranded DNA fragments was selected. DNA fragments were end damage repaired and ligated with universal hairpin adapters. Subsequent steps were followed as per the manufacture’s instruction to prepare SMRTbell library. The library was sequenced in PacBio SEQUEL instrument^75^.

PacBio reads were assembled using HGAP4/Falcon of WGS-Assembler 8.2^76–81^. And then we recorrect the genome with software Pilon using previous illumina data or Quiver using Pacbio reads. The Prodigal^82^/Augustus^83^ gene-finding software has been used for finding coding genes. The coding genes were annotated with National Center for Biotechnology Information (NCBI) NR database by BLAST. Then the functions of genes were annotated by GO^84^ (Gene Ontology) database, and the pathways were annotated using KEGG^85^ (Kyoto Encyclopedia of Genes and Genomes) database.

### Mining the specific targets of *Lactobacillus plantarum* 124 through pan-genome

All complete, closed genome sequences available in GenBank for *Lactobacillus plantarum* were downloaded and included in the analysis for generating the species pangenomes^86^. Based on this analysis, a list of unique genes for each species, i.e., present in one species and absent in the other one, was generated. In order to confirm the specificities of these genes, a search of the unique genes listed for each species was performed. Firstly, against all *Lactobacillus plantarum* genomes and, secondly, against the NCBI database for prokaryotes. For validation of the suggested unique genes as species biomarkers, 227 isolated strains of *Lactobacillus* and non-*Lactobacillus* were screened by PCR for the presence of the unique gene markers, determined from the pangenome analysis (Table S7).

### Animal experiments and determination of oxidoreductase activity

Institute of Microbiology, Guangdong Academy of Sciences for animal experiment ethics approval number: GT-IACUC201909026. Kunming mice (SPF grade) were obtained from the Southern Medical University (Guangdong, China) and housed under a 12-h light/dark cycle in the gnotobiotic facilities. All mice were fed with sterile food and water *ad libitum*. The control group received a subcutaneous injection of normal saline (200 mg/kg/day), and 0.2 mL of normal saline was gavaged daily. The model group was injected subcutaneously with D-galactose (200 mg/kg/day), and 0.2 mL normal saline was gavaged daily. For the test group, D-galactose (200 mg/kg/day) was injected subcutaneously, and 0.2 mL of 1.01 ± 0.05 × l0^9^ CFU/mL A/B bacterial solution was gavaged daily. Strain A consisted of *Lactobacillus plantarum* 124 (test strain) and strain B consisted of *Akkermansia muciniphila* strain ATCC BAA-835 (control strain). The positive drug Vc group received D-galactose (200 mg/kg/day) injected subcutaneously, and the antioxidant Vc (200 mg/kg/day) was gavaged daily. The feeding cycle was 9 weeks. Liver and kidney tissue homogenates were prepared for analysis for oxidative stress markers such as glutathione peroxidase (GSH-Px), superoxide dismutase (SOD), thioredoxin reductase (TrxR), total antioxidant capacity levels (T-AOC), malondialdehyde (MDA), carbonyl, and endotoxin (ET).

### Morphological observations

After an animal was sacrificed, its liver, kidney, and small intestine were isolated. The tissues and organs were fixed with 10% formalin, embedded in conventional paraffin, sectioned to a thickness of 4–5 μm, stained with haematoxylin and eosin (HE), and the results read by pathology professionals. Sections were assessed according to the severity of the lesion: mild (+), moderate (++), severe (+++), and no diseased tissue (−).

### Genes expression analysis

Total RNA was extracted from mice liver and kidney using an RNeasy mini kit (Huangshi Yanke Biotechnology Co., LTD, Hubei, China) according to manufacturer’s instructions. Quantitative RT-PCR was performed using SYBR Premix Ex Taq (Huangshi Yanke Biotechnology Co., LTD, Hubei, China) with the Applied Biosystems 7500 Fast Real-Time PCR System. The calculation of mRNA expression was performed by the 2^−ΔΔCT^ method using the geometric mean of the housekeeping genes *GSH-Px*, *SOD*, *TrxR*, and nuclear factor-erythroid 2-related factor 2 (*Nrf2*). The genes and primer sequences are listed in Table S8. Comparisons between groups were performed using one-way ANOVA with *n* = 6–9 per group. All results are presented as means ± SD of the biological replicates.

### Mass Spectrometry

Serum samples were collected from model and LP animals (*n* = 6 per group), acetonitrile was used to extract metabolites by protein precipitation. Untargeted metabolomics profiling was performed using liquid chromatography coupled to a Thermo Scientific High-Field Qexactive mass spectrometer (HILIC/ESI+, C18/ESI-, 85-1,275 m/z, 120k resolution). Spectral features (m/z, retention time) corresponding to identified and uncharacterized metabolites were integrated and aligned using apLCMS/xMSanalyzer software. Metaboanalyst^87^ was used for statistical analysis and Mummichog software^88^ was used for pathway enrichment analysis and verified by retention time, m/z, and MS/MS by authenticated standards. Candidate molecule were identified following ion dissociation experiments on a Thermo Scientific Fusion mass spectrometer, and spectral library was matched to the mzCloud, mzVault, and MassList library using Compound Discoverer 3.0.

### Statistics

According to the different data, statistical analysis between groups was conducted using the Wilcoxon’s rank sum test, Student’s *t*-test, or one-way ANOVA analysis of variance and expressed as mean ± SD. The Shannon index at the genera level was calculated with QIIME (v1.9.1). R software (v2.15.3) was used to analyse the difference between groups of alpha diversity index and beta diversity index. *p* < 0.05 indicates statistical significance. Statistical analyses and data visualisation were performed using R software (v2.15.3) with the WGCNA, stats, and ggplot2 software packages.

### Reporting summary

Further information on research design is available in the Nature Research Reporting Summary linked to this article.

## Supplementary information

Supplementary Information

Reporting Summary

Supplementary Tables

## Data Availability

The sequence data for all samples have been deposited in the NCBI Sequence Read Archive (SRA) under accession code BIOProject: PRJNA895352. Other data that support the findings of this study are available within the paper and its Supplementary Information files or from the corresponding author upon reasonable request.

## References

[1] Ctoi AF (2020). Gut microbiota and aging-A focus on centenarians. Biochim. Biophys. Acta Mol. Basis Dis..

[2] Wu L (2022). Integrated Multi-omics For Novel Aging Biomarkers And Antiaging Targets. Biomolecules.

[3] Finlay BB, Pettersson S, Melby MK, Bosch TCG (2019). The microbiome mediates environmental effects on aging. BioEssays: N. Rev. Mol., Cell. Dev. Biol..

[4] Wu, L. et al. Bacterial diversity and community in regional water microbiota between different towns in world’s longevity township Jiaoling. *China Diversity* **13**, 361 (2021).

[5] Han B (2018). Microbial genetic composition tunes host longevity. Cell.

[6] Wu L (2021). Metagenomics-based analysis of the age-related cumulative effect of antibiotic resistance genes in gut microbiota. Antibiotics.

[7] Wu L (2022). Washed microbiota transplantation improves patients with high blood glucose in South China. Front. Endocrinol..

[8] Wu L (2022). Washed microbiota transplantation improves patients with metabolic syndrome in South China. Front. Cell. Infect. Microbiol..

[9] Nagpal R (2018). Gut microbiome and aging: physiological and mechanistic insights. Nutr. Health Aging.

[10] Smith P (2017). Regulation of life span by the gut microbiota in the short-lived African turquoise killifish. eLife.

[11] Iatsenko I, Boquete JP, Lemaitre B (2018). Microbiota-derived lactate activates production of reactive oxygen species by the intestinal NADPH oxidase nox and shortens drosophila lifespan. Immunity.

[12] Debebe T (2017). Unraveling the gut microbiome of the long-lived naked mole-rat. Sci. Rep..

[13] Anderson KE (2018). The queen’s gut refines with age: longevity phenotypes in a social insect model. Microbiome.

[14] Wang F (2015). Gut microbiota community and its assembly associated with age and diet in Chinese centenarians. J. Microbiol Biotechnol..

[15] Rampelli S (2013). Functional metagenomic profiling of intestinal microbiome in extreme ageing. Aging.

[16] Wu L (2019). A cross-sectional study of compositional and functional profiles of gut microbiota in Sardinian Centenarians. mSystems.

[17] Sato Y (2021). Novel bile acid biosynthetic pathways are enriched in the microbiome of centenarians. Nature.

[18] Bilen M (2018). The contribution of culturomics to the repertoire of isolated human bacterial and archaeal species. Microbiome.

[19] Lagier JC (2015). The rebirth of culture in microbiology through the example of culturomics to study human gut microbiota. Clin. Microbiol. Rev..

[20] Lagier JC (2012). Microbial culturomics: paradigm shift in the human gut microbiome study. Clin. Microbiol. Infect..

[21] Dubourg G (2014). Culturomics and pyrosequencing evidence of the reduction in gut microbiota diversity in patients with broad-spectrum antibiotics. Int. J. Antimicrob. Agents.

[22] Sepp E (2022). Comparative analysis of gut microbiota in centenarians and young people: impact of eating habits and childhood living environment. Front Cell Infect. Microbiol.

[23] Bian G (2017). The gut microbiota of healthy aged Chinese is similar to that of the healthy young. mSphere.

[24] Shade A (2017). Diversity is the question, not the answer. ISME J..

[25] Biagi E (2016). Gut microbiota and extreme longevity. Curr. Biol..

[26] Ren M, Li H, Fu Z, Li Q (2021). Succession analysis of gut microbiota structure of participants from long-lived families in Hechi, Guangxi, China. Microorganisms.

[27] Palmas V (2022). Gut microbiota markers and dietary habits associated with extreme longevity in healthy Sardinian Centenarians. Nutrients.

[28] Bárcena C (2019). Healthspan and lifespan extension by fecal microbiota transplantation into progeroid mice. Nat. Med..

[29] Paone P, Cani PD (2020). Mucus barrier, mucins and gut microbiota: the expected slimy partners?. Gut.

[30] Plovier H (2017). A purified membrane protein from *Akkermansia muciniphila* or the pasteurized bacterium improves metabolism in obese and diabetic mice. Nat. Med..

[31] Depommier C (2019). Supplementation with *Akkermansia muciniphila* in overweight and obese human volunteers: a proof-of-concept exploratory study. Nat. Med..

[32] Jie Z (2017). The gut microbiome in atherosclerotic cardiovascular disease. Nat. Commun..

[33] Jie Z (2021). A transomic cohort as a reference point for promoting a healthy human gut microbiome. Med. Microecol..

[34] Bonder, M. J. The interplay between genetics, the microbiome, DNA methylation & gene expression. PhD thesis, University of Groningen (2017).

[35] Su L (2013). Tnfr2 activates MlcK-dependent tight junction dysregulation to cause apoptosis-mediated barrier loss and experimental colitis. Gastroenterology.

[36] Anitra CC, Silvia M (2017). Vitamin C, and immune function. Nutrients.

[37] Mario F (2019). Vitamin C content in fruits: Biosynthesis and regulation. Front. Plant Sci..

[38] Mirza H (2019). Regulation of ascorbate-glutathione pathway in mitigating oxidative damage in plants under abiotic stress. Antioxidants.

[39] Thursby E, Juge N (2017). Introduction to the human gut microbiota. Biochem. J..

[40] Biagi E (2017). The gut microbiota of centenarians: signatures of longevity in the gut microbiota profile. Mech. Ageing Dev..

[41] Degen LP, Phillips SF (1996). Variability of gastrointestinal transit in healthy women and men. Gut.

[42] Graff J, Brinch K, Madsen JL (2001). Gastrointestinal mean transit times in young and middle-aged healthy subjects. Clin. Physiol..

[43] Roager HM (2016). Colonic transit time is related to bacterial metabolism and mucosal turnover in the gut. Nat. Microbiol..

[44] Hou Y (2019). Ageing as a risk factor for neurodegenerative disease. Nat. Rev. Neurol..

[45] Wu L (2022). Integrated multi-omics for novel aging biomarkers and antiaging targets. Biomolecules.

[46] Van Der Lugt B (2019). *Akkermansia muciniphila* ameliorates the age-related decline in colonic mucus thickness and attenuates immune activation in accelerated aging *Ercc1*^−/Δ7^ mice. Immun. Ageing.

[47] Samuel BS (2007). Genomic and metabolic adaptations of *Methanobrevibacter smithii* to the human gut. Proc. Natl Acad. Sci. USA.

[48] Triantafyllou K, Chang C, Pimentel M (2014). Methanogens, methane, and gastrointestinal motility. J. Neurogastroenterol. Motil..

[49] Li CY (2022). Deep insights into the gut microbial community of extreme longevity in south Chinese centenarians by ultra-deep metagenomics and large-scale culturomics. npj Biofilms Microbi.

[50] Simone R (2020). Shotgun metagenomics of gut microbiota in humans with up to extreme longevity and the increasing role of xenobiotic degradation. mSystems.

[51] Rampelli S (2015). Metagenome sequencing of the Hadza hunter-gatherer gut microbiota. Curr. Biol..

[52] Ho JTK, Chan GCF, Li JCB (2015). Systemic effects of gut microbiota and its relationship with disease and modulation. BMC Immunol..

[53] Kim BS (2019). Comparison of the gut microbiota of centenarians in longevity villages of South Korea with those of other age Groups. J. Microbiol Biotechnol..

[54] Martinez-Guryn K, Leone V, Chang EB (2019). Regional diversity of the gastrointestinal microbiome. Cell Host Microbe.

[55] Qin J (2012). A metagenome-wide association study of gut microbiota in type 2 diabetes. Nature.

[56] Aßhauer K (2015). Tax4Fun: predicting functional profiles from metagenomic 16S rRNA data. Bioinformatics.

[57] Martin M (2011). Cutadapt removes adapter sequences from high-throughput sequencing reads. Embnet J..

[58] Rognes T (2016). VSEARCH: a versatile open source tool for metagenomics. PeerJ.

[59] Haas BJ (2011). Chimeric 16S rRNA sequence formation and detection in Sanger and 454-pyrosequenced PCR amplicons. Genome Res.

[60] Wang Q (2007). Naive Bayesian classifier for rapid assignment of rRNA sequences into the new bacterial taxonomy. Appl. Environ. Microbiol.

[61] Edgar RC (2013). UPARSE: highly accurate OTU sequences from microbial amplicon reads. Nat. Methods.

[62] Segata N (2011). Metagenomic biomarker discovery, and explanation. Genome Biol..

[63] Feng Q (2015). Gut microbiome development along the colorectal adenoma-carcinoma sequence. Nat. Commun..

[64] Li J (2014). An integrated catalog of reference genes in the human gut microbiome. Nat. Biotechnol..

[65] Kanehisa M (2006). From genomics to chemical genomics: new developments in KEGG. Nucleic Acids Res.

[66] Kanehisa M (2017). KEGG: new perspectives on genomes, pathways, diseases, and drugs. Nucleic Acids Res.

[67] Jaime HC (2016). eggNOG 4.5: a hierarchical orthology framework with improved functional annotations for eukaryotic, prokaryotic and viral sequences. Nucleic Acids Res.

[68] Schiebenhoefer H (2020). A complete and flexible workflow for metaproteomics data analysis based on MetaProteomeAnalyzer and Prophane. Nat. Protoc..

[69] Shimada K (1992). Antioxidative properties of xanthan on the anti-oxidation of soybean oil in cyclodextrin emulsion. J. Agric. Food Chem..

[70] Oyaizu M (1986). Antioxidative activities of browning reaction prepared from glucosamine. Jpn. J. Nutr..

[71] He ZS (2004). Photometric determination of hydroxyl free radical in Fenton system by brilliant green. Am. J. Chin. Clin. Med..

[72] Amanatidou A (2001). Antioxidative properties of lactobacillus sake upon exposure to elevated oxygen concentrations. FEMS Microbiol. Lett..

[73] Gao D, Gao Z, Zhu G (2013). Antioxidant effects of Lactobacillus plantarum via activation of transcription factor Nrf2. Food Funct..

[74] Kullisaar T (2003). Antioxidative probiotic fermented goats’milk decreases oxidative stress-mediated atherogenicity in human subjects. Br. J. Nutr..

[75] Mccarthy A (2010). Third generation DNA sequencing: pacific biosciences' single molecule real time technology. Chem. Biol..

[76] Myers EW (2000). A whole-genome assembly of Drosophila. Science.

[77] Venter JC (2001). The sequence of the human genome. Science.

[78] Istrail S (2004). Whole-genome shotgun assembly and comparison of human genome assemblies. Proc. Natl Acad. Sci. USA.

[79] Levy S (2007). The diploid genome sequence of an individual human. PLoS Biol..

[80] Goldberg SMD (2006). A Sanger/pyrosequencing hybrid approach for the generation of high-quality draft assemblies of marine microbial genomes. Proc. Natl Acad. Sci. USA.

[81] Berlin K (2015). Assembling large genomes with single-molecule sequencing and locality-sensitive hashing. Nat. biotechnol..

[82] Delcher AL (2007). Identifying bacterial genes and endosymbiont DNA with Glimmer. Bioinformatics.

[83] Stanke M (2006). Gene prediction in eukaryotes with a generalized hidden Markov model that uses hints from external sources. BMC Bioinforma..

[84] Gene OC (2004). The Gene Ontology (GO) database and informatics resource. Nucleic Acids Res.

[85] Kanehisa M, Susumu G (2000). KEGG: kyoto encyclopedia of genes and genomes. Nucleic Acids Res.

[86] Gonzales-Siles L (2020). A pangenome approach for discerning species-unique gene markers for identifications of *Streptococcus pneumoniae* and *Streptococcus pseudopneumoniae*. Front. Cell Infect. Mi..

[87] Xia J, Wishart DS (2016). Using MetaboAnalyst 3.0 for comprehensive metabolomics data analysis. Curr. Protoc. Bioinforma..

[88] Li S (2013). Predicting network activity from high throughput metabolomics. PLoS Comput. Biol..

