# Supplementary material for: Gut microbiota as an antioxidant system in centenarians associated with high antioxidant activities of gut-resident Lactobacillus
Source: NPJ Biofilms Microbiomes. 2022 Dec 24;8:102. doi: 10.1038/s41522-022-00366-0 (PMC9789086; doi:10.1038/s41522-022-00366-0)
Supplement: Supplementary file 1 — Supplementary Information [file 41522_2022_366_MOESM1_ESM.pdf]

The Supplementary materials include:

Supplementary Figures:

Supplementary Figure 1 Species composition characteristics of different taxonomic levels of gut microbiota in different age groups. (**a-d**) Comparison of alpha-diversity indices (Observed species, Chao1, Shannon and ACE index index) between six groups using Wilcoxon rank-sum test. * indicates $p < 0.05$; ** indicates $p < 0.01$; *** indicates $p < 0.001$. (**e**) Principal coordinate analysis based on unweighted UniFrac distances revealed that the Y20, Y40, Y60 bacterial communities clustered separately from Y80, Y100, Y120 bacterial communities, which were more similar to each other. Each circle represents a single sample, coloured by group. The eigenvalues of axe Principal coordinate (PC)1 and PC2 were 10.65% and 5.41%, respectively. (**f**) Bar graph of the relative abundance of the first 10 species of gut microbiota in different age groups at the phylum level. (**g,h**) Clustering heat map of the composition abundance of the top 35 species in the gut microbiota of different age groups at the family and genus level.

Supplementary Figure 2 Analysis of different species between groups. Linear discriminant analysis effect size identified the most differentially abundant taxa between the two groups. Y120-enriched taxa are indicated with a positive LDA score, and taxa enriched in Y20, Y40, Y60,Y80 controls have a negative score. Only taxa meeting an LDA significant threshold of >3 are shown.

Supplementary Figure 3 Age-related gut functional characteristics of xenobiotics biodegradation and metabolism and oxidoreductase. (**a**) Box plot showing the changes of gut microbial function xenobiotics biodegradation and metabolism (aminobenzoate, atrazine, benzoate, caprolactam, chloroalkane and chloroalkene, chlorocyclohexane and chlorobenzene, dioxin, ethylbenzene, naphthalene, nitrotoluene, polycyclic aromatic hydrocarbon, styrene, toluene, and xylene degradation) with age groups. (**b**) Box plot showing the changes of oxidoreductase (NADH ubiquinone plastoquinone oxidoreductase, thioredoxin, glutathione

peroxidase, and heme oxygenase) with age groups. * indicates $p < 0.05$; ** indicates $p < 0.01$. Statistical analyses were performed using two-sided Wilcoxon rank sum tests. For all box and whisker plots, the center line represents median. The bounds of box represent the first and third quartiles. The upper whisker extends from the hinge to the largest value no further than 1.5 * interquartile range (IQR) from the hinge. The lower whisker extends from the hinge to the smallest value at most 1.5 * IQR of the hinge.

Supplementary Figure 4 Whole-genome and pan-genome analysis of *Lactobacillus plantarum* 124. (**a**) Isolation of strains. (**b,c**) The bar graph showing the KEGG pathway annotation. The functions of genes were annotated by GO (Gene Ontology) database, and the pathways were annotated using KEGG (Kyoto Encyclopedia of Genes and Genomes) database. (**d**) PCR nucleic acid gel electrophoresis for specific target sequence diagram. For validation of the suggested unique genes as species biomarkers, 227 isolated strains of *Lactobacillus* and non-*Lactobacillus* were screened by PCR for the presence of the unique gene markers, determined from the pangenome analysis. All gels derive from the same experiment and that they were
processed in parallel.

Supplementary Figure 5 *Lactobacillus plantarum* 124 and *Akkermansia* regulate the composition and function of intestinal flora in oxidatively damaged mice. (**a-c**) Effect of *Lactobacillus plantarum* 124 and *Akkermansia* on the species abundance of mice intestinal flora (At the eighth week, A6, control group; B6 model group; C6, antibiotic group; D6, LP group; E6, antibiotic + LP group; F6, AKK group; G6, antibiotic + AKK; H6, LP + AKK group; I6 , Antibiotic+LP+AKK group; K6, Vc group). (**d**) Correlation diagram of different metabolites (the pos is the positive ion mode, the neg is the negative ion mode). (**e**) KEGG pathway enrichment bubble chart. (**f-i**) PLS-DA get scatter plot and sort verification plot. The scatter plot, R2Y represents the interpretation rate of the model, and Q2Y is used to evaluate the predictive ability of the PLS-DA model. And when R2Y is greater than Q2Y, it means

that the model is well established. For ranking test, the abscissa represents the correlation between the Y of the random grouping and the original group Y, and the ordinate represents the scores of R2 and Q2. (**j**) Screening of *Lactobacillus plantarum* 124 antioxidant capacity. Scavenging rates of DPPH, reducing activities L-cysteine equivalent (μmol / L), scavenging rates of hydroxyl free radical (·OH), chelating rates ferrous ion (Fe^{2+}), scavenging rates of superoxide anion ($\cdot O^{2-}$), inhibition of lipid peroxidation. (**k**) Changes in mice body weight. (**l**) Organ index of mice liver and kidney. Different letters (a, b, c) in the figure indicate significant differences between groups ($p < 0.05$, one-way ANOVA analysis), and the same letters indicate no significant differences. Statistical analysis between groups was expressed as mean ± SD, error bars were standard deviation.

Supplementary Tables:

Supplementary Table 1 Description and summary statistics of the Jiaoling cohort (n=247).

Supplementary Table 2 Preliminary screening of antioxidant strains.

Supplementary Table 3 Re-screening of antioxidant strains.

Supplementary Table 4 Specific molecular target sequence 124_03212 of LP124.

Supplementary Table 5 Metabolomics analysis of LP 124 (negative mode).

Supplementary Table 6 Metabolomics analysis of LP 124 (positive mode).

Supplementary Table 7 Test results for specific targets.

Supplementary Table 8 The gene and primer sequences in RT-PCR analysis.

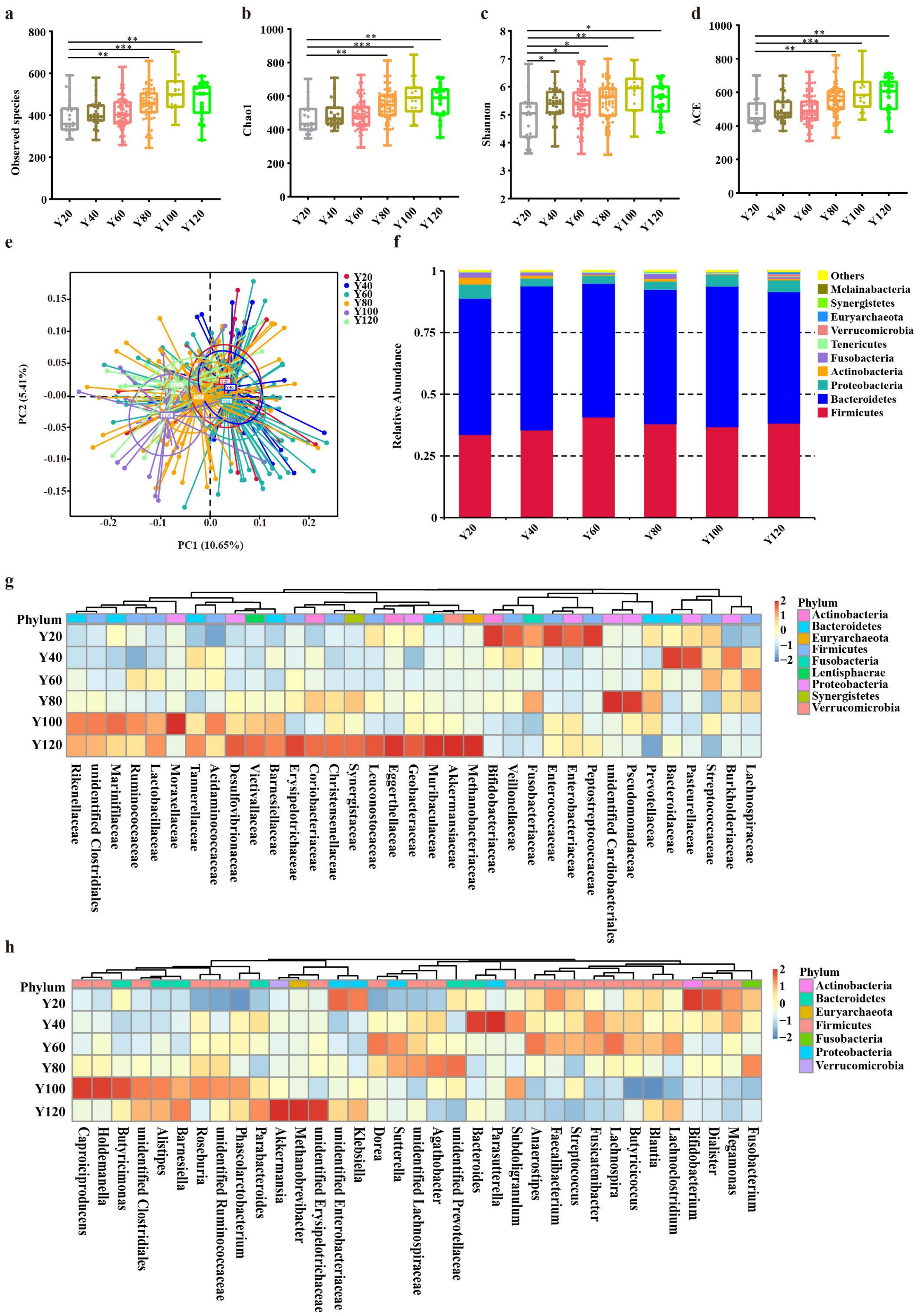

Supplementary Figure 1 Species composition characteristics of different taxonomic levels of gut microbiota in different age groups. (**a-d**) Comparison of alpha-diversity indices (Observed species, Chao1, Shannon and ACE index index) between six groups using Wilcoxon rank-sum test. * indicates $p < 0.05$; ** indicates $p < 0.01$; *** indicates $p < 0.001$. (**e**) Principal coordinate analysis based on unweighted UniFrac distances revealed that the Y20, Y40, Y60 bacterial

communities clustered separately from Y80, Y100, Y120 bacterial communities, which were more similar to each other. Each circle represents a single sample, coloured by group. The eigenvalues of axe Principal coordinate (PC)1 and PC2 were 10.65% and 5.41%, respectively. (**f**) Bar graph of the relative abundance of the first 10 species of gut microbiota in different age groups at the phylum level. (**g,h**) Clustering heat map of the composition abundance of the top 35 species in the gut microbiota of different age groups at the family and genus level.

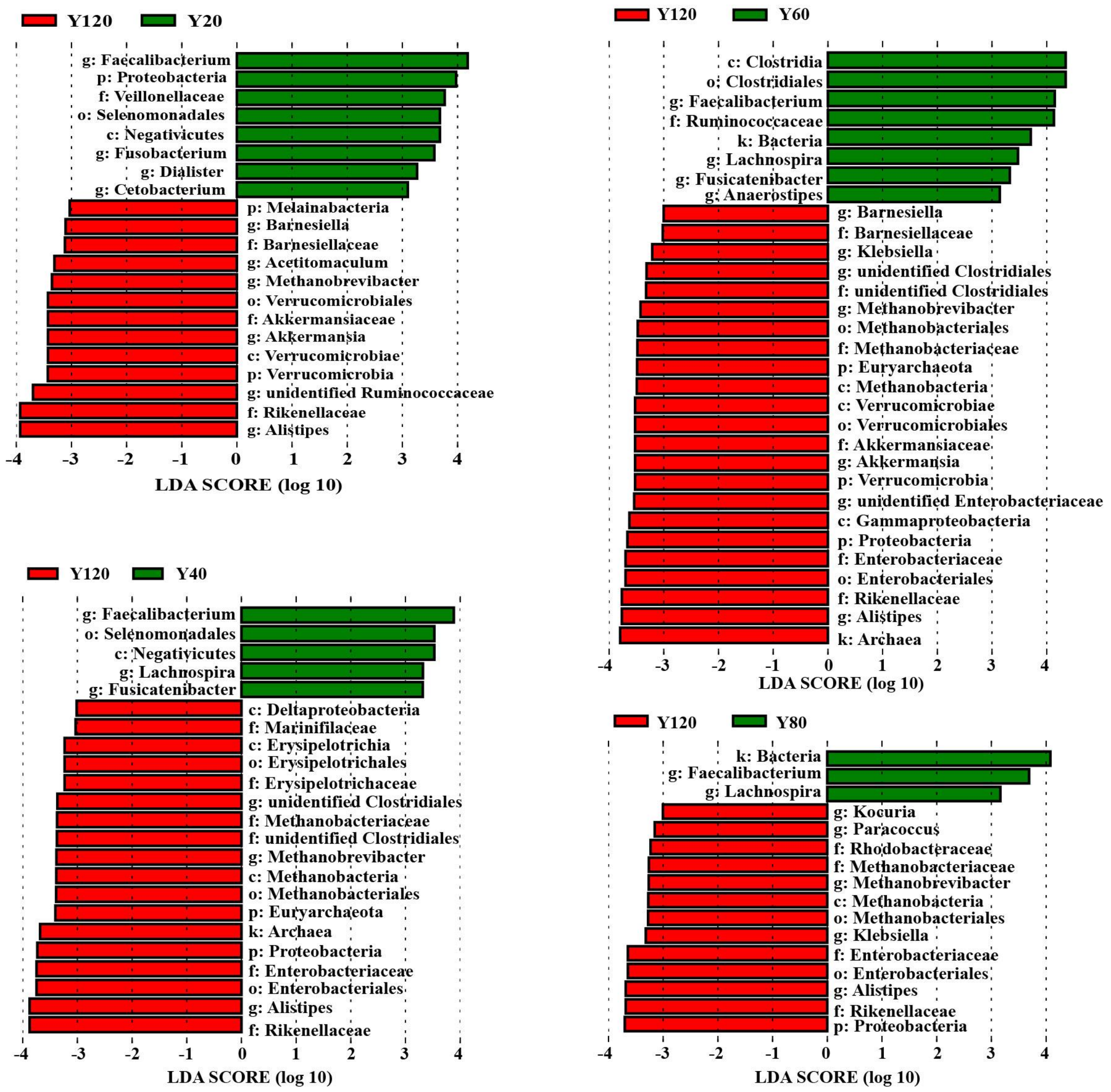

Supplementary Figure 2 Analysis of different species between groups. Linear discriminant analysis effect size identified the most differentially abundant taxa between the two groups. Y120-enriched taxa are indicated with a positive LDA score, and taxa enriched in Y20, Y40, Y60,Y80 controls have a negative score. Only taxa meeting an LDA significant threshold of >3 are shown.

a

Xenobiotics biodegradation and metabolism

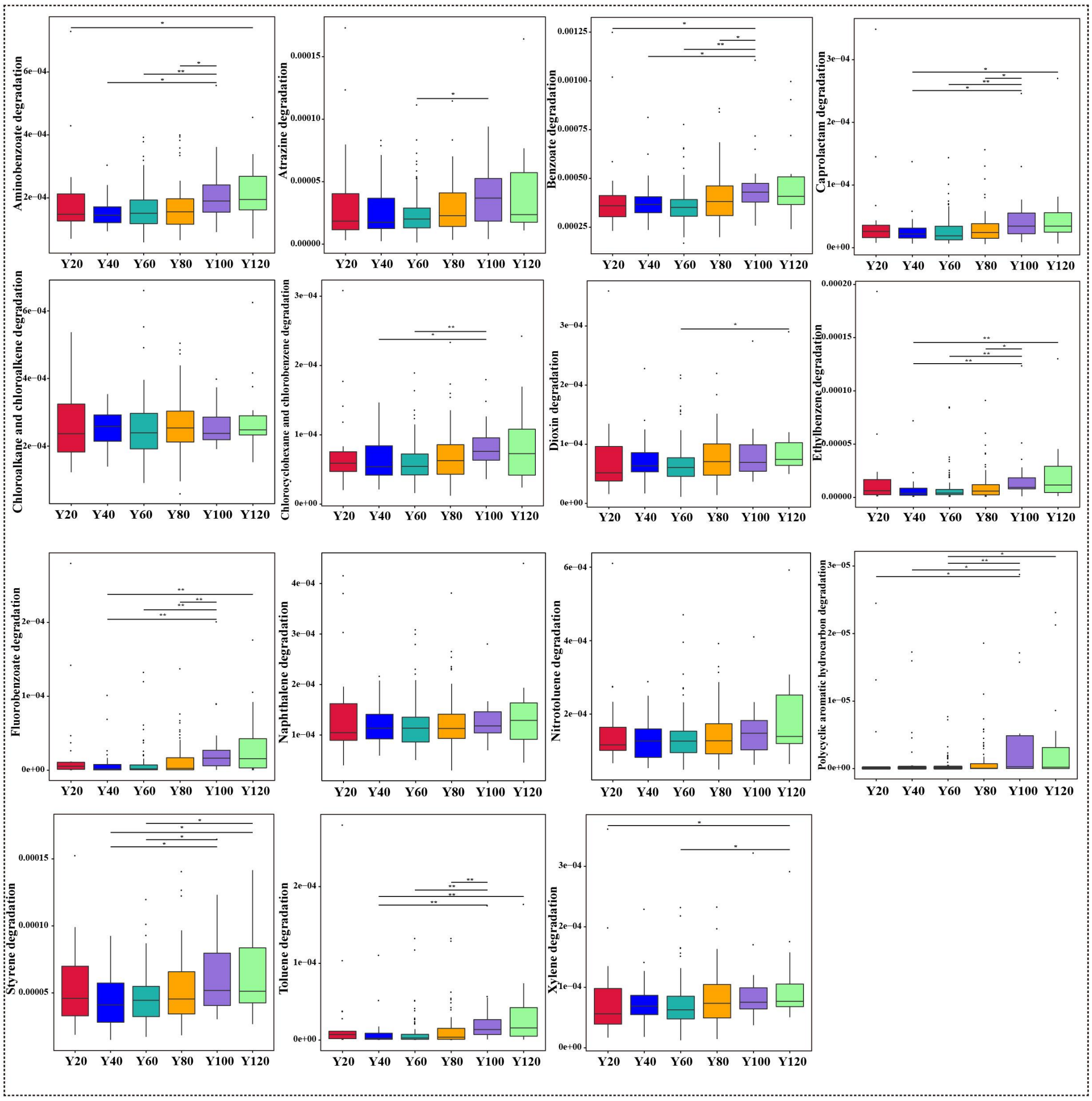

b

Oxidoreductase

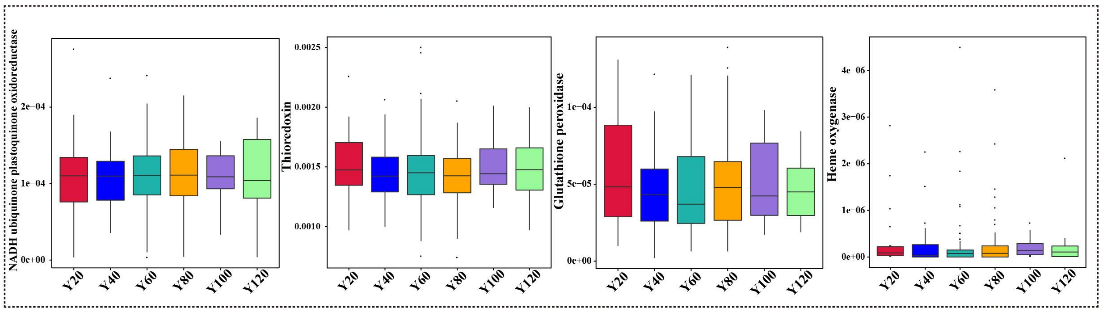

Supplementary Figure 3 Age-related gut functional characteristics of xenobiotics biodegradation and metabolism and oxidoreductase. (**a**) Box plot showing the changes of gut microbial function xenobiotics biodegradation and metabolism (aminobenzoate, atrazine, benzoate, caprolactam, chloroalkane and chloroalkene, chlorocyclohexane and chlorobenzene, dioxin, ethylbenzene, naphthalene, nitrotoluene, polycyclic aromatic hydrocarbon, styrene, toluene, and xylene degradation) with age groups. (**b**) Box plot showing the changes of oxidoreductase (NADH

ubiquinone plastoquinone oxidoreductase, thioredoxin, glutathione peroxidase, and heme oxygenase) with age groups. * indicates $p < 0.05$; ** indicates $p < 0.01$. Statistical analyses were performed using two-sided Wilcoxon rank sum tests. For all box and whisker plots, the center line represents median. The bounds of box represent the first and third quartiles. The upper whisker extends from the hinge to the largest value no further than 1.5 * interquartile range (IQR) from the hinge. The lower whisker extends from the hinge to the smallest value at most 1.5 * IQR of the hinge.

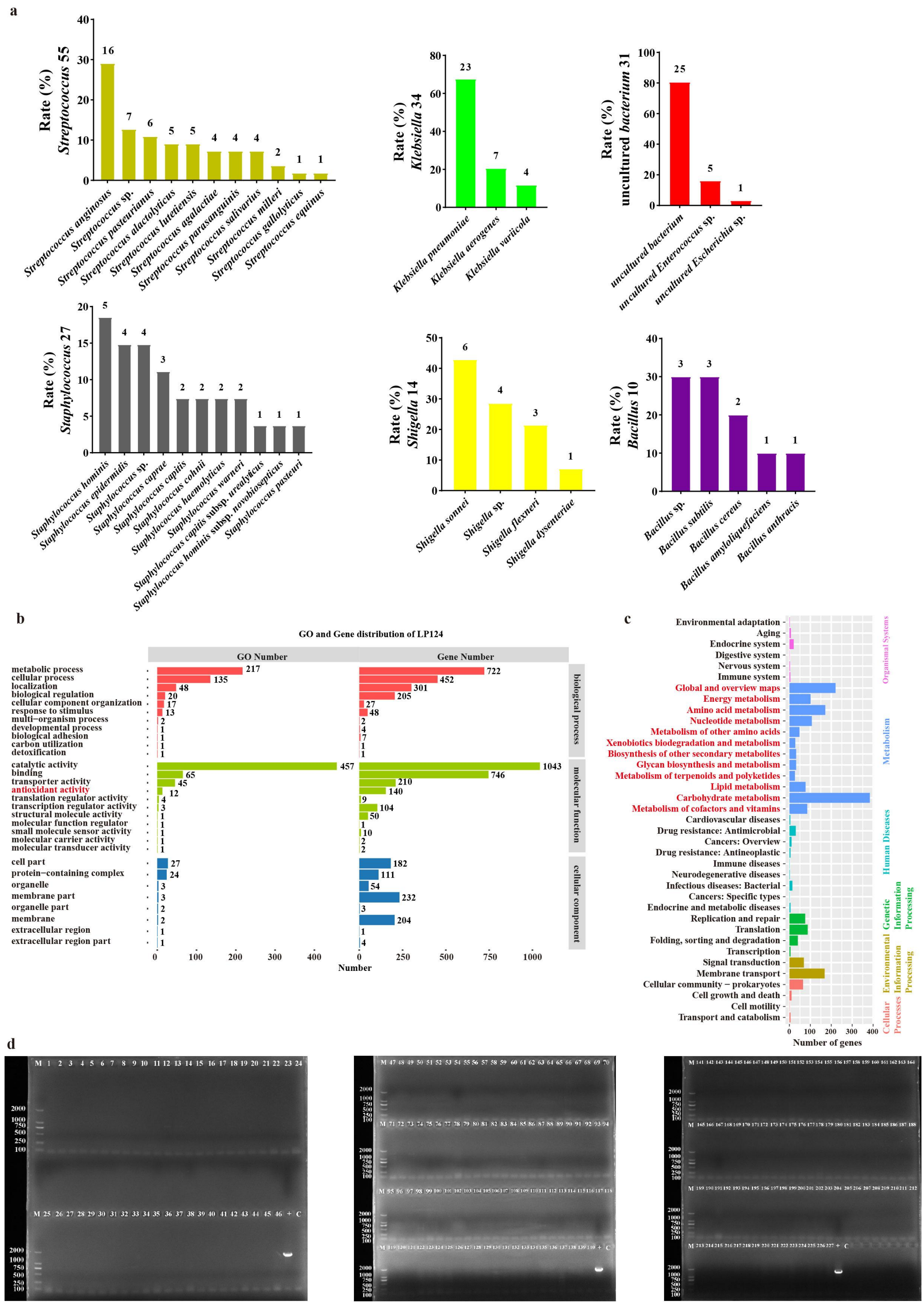

Supplementary Figure 4 Whole-genome and pan-genome analysis of *Lactobacillus plantarum* 124. (**a**) Isolation of strains. (**b,c**) The bar graph showing the KEGG pathway annotation. The functions of genes were annotated by GO (Gene Ontology) database, and the pathways were annotated using KEGG (Kyoto Encyclopedia of Genes and Genomes) database. (**d**) PCR nucleic acid gel electrophoresis for specific target sequence diagram. For validation of the suggested unique genes as species biomarkers, 227 isolated strains of *Lactobacillus* and non-*Lactobacillus*

were screened by PCR for the presence of the unique gene markers, determined from the pangenome analysis. All gels derive from the same experiment and that they were processed in parallel.

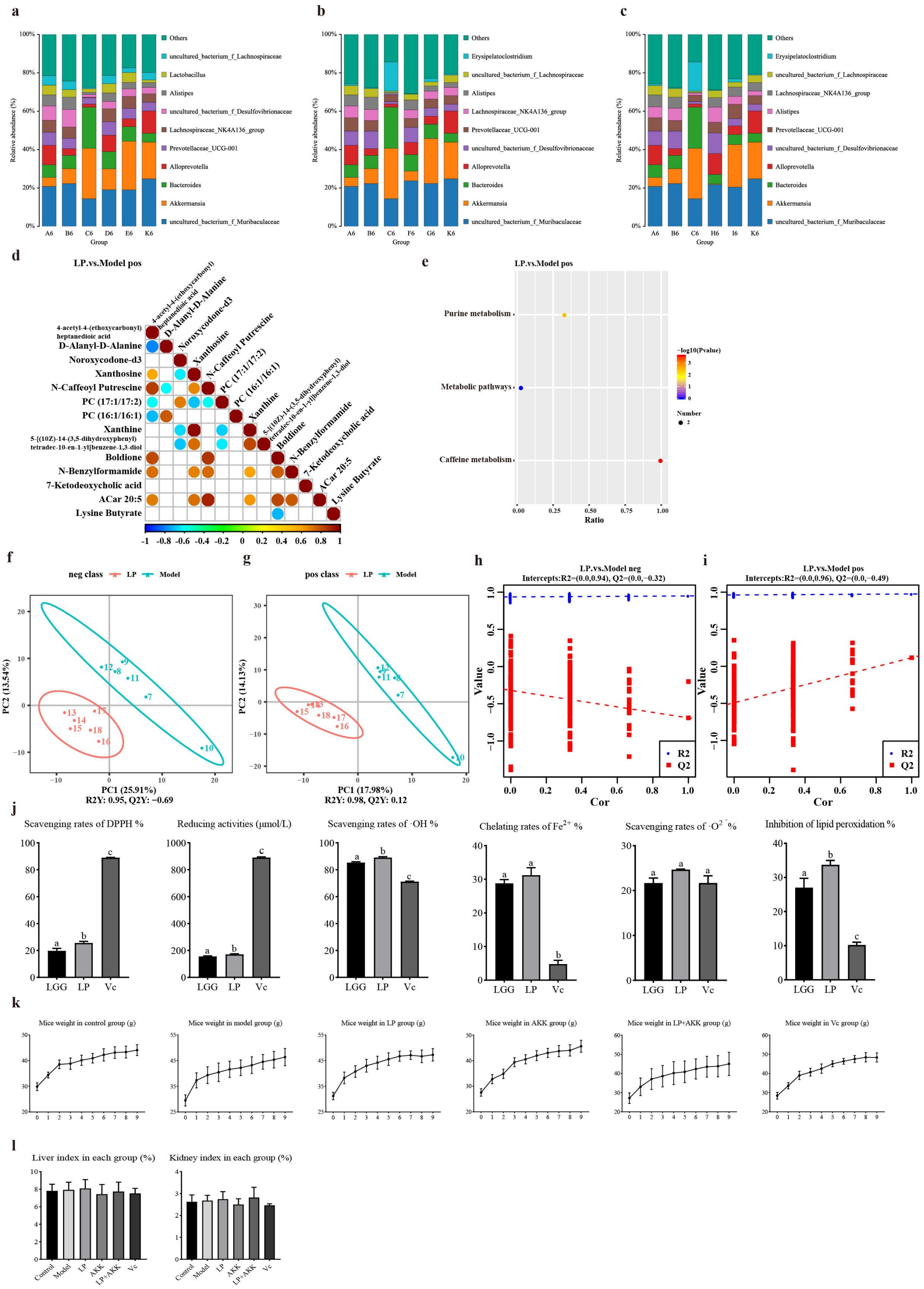

Supplementary Figure 5 ***Lactobacillus plantarum*** **124 and** ***Akkermansia*** **regulate the composition and function of intestinal flora in oxidatively damaged mice.** (**a-c**) Effect of *Lactobacillus plantarum* 124 and *Akkermansia* on the species abundance of mice intestinal flora (At the eighth week, A6, control group; B6 model group; C6, antibiotic group; D6, LP group; E6, antibiotic + LP group; F6, AKK group; G6, antibiotic + AKK; H6, LP + AKK group; I6 , Antibiotic+LP+AKK group; K6, Vc group). (**d**) Correlation diagram of different metabolites (the

pos is the positive ion mode, the neg is the negative ion mode). (**e**) KEGG pathway enrichment bubble chart. (**f-i**) PLS-DA get scatter plot and sort verification plot. The scatter plot, R2Y represents the interpretation rate of the model, and Q2Y is used to evaluate the predictive ability of the PLS-DA model. And when R2Y is greater than Q2Y, it means that the model is well established. For ranking test, the abscissa represents the correlation between the Y of the random grouping and the original group Y, and the ordinate represents the scores of R2 and Q2. (**j**) Screening of *Lactobacillus plantarum* 124 antioxidant capacity. Scavenging rates of DPPH, reducing activities L-cysteine equivalent (μmol / L), scavenging rates of hydroxyl free radical (·OH), chelating rates ferrous ion (Fe^{2+}), scavenging rates of superoxide anion (·O^{2-}), inhibition of lipid peroxidation. (**k**) Changes in mice body weight. (**l**) Organ index of mice liver and kidney. Different letters (a, b, c) in the figure indicate significant differences between groups ($p < 0.05$, one-way ANOVA analysis), and the same letters indicate no significant differences. Statistical analysis between groups was expressed as mean ± SD, error bars were standard deviation.
