# Supplementary material for: Gut microbiota as an antioxidant system in centenarians associated with high antioxidant activities of gut-resident Lactobacillus
Source: NPJ Biofilms Microbiomes. 2022 Dec 24;8:102. doi: 10.1038/s41522-022-00366-0 (PMC9789086; doi:10.1038/s41522-022-00366-0)
Supplement: Supplementary file 2 — Reporting Summary [file 41522_2022_366_MOESM2_ESM.pdf]

nature portfolio

Corresponding author(s): Qingping Wu

Last updated by author(s): Nov 28, 2022

Reporting Summary

Nature Portfolio wishes to improve the reproducibility of the work that we publish. This form provides structure for consistency and transparency in reporting. For further information on Nature Portfolio policies, see our Editorial Policies and the Editorial Policy Checklist.

Statistics

For all statistical analyses, confirm that the following items are present in the figure legend, table legend, main text, or Methods section.

n/a	Confirmed	
☐	☒	The exact sample size (*n*) for each experimental group/condition, given as a discrete number and unit of measurement
☐	☒	A statement on whether measurements were taken from distinct samples or whether the same sample was measured repeatedly
☐	☒	The statistical test(s) used AND whether they are one- or two-sided<br>*Only common tests should be described solely by name; describe more complex techniques in the Methods section.*
☒	☐	A description of all covariates tested
☒	☐	A description of any assumptions or corrections, such as tests of normality and adjustment for multiple comparisons
☐	☒	A full description of the statistical parameters including central tendency (e.g. means) or other basic estimates (e.g. regression coefficient) AND variation (e.g. standard deviation) or associated estimates of uncertainty (e.g. confidence intervals)
☐	☒	For null hypothesis testing, the test statistic (e.g. *F*, *t*, *r*) with confidence intervals, effect sizes, degrees of freedom and *P* value noted<br>*Give P values as exact values whenever suitable.*
☒	☐	For Bayesian analysis, information on the choice of priors and Markov chain Monte Carlo settings
☒	☐	For hierarchical and complex designs, identification of the appropriate level for tests and full reporting of outcomes
☒	☐	Estimates of effect sizes (e.g. Cohen's *d*, Pearson's *r*), indicating how they were calculated

Our web collection on statistics for biologists contains articles on many of the points above.

Software and code

Policy information about availability of computer code

Data collection	Libraries with different indices were multiplexed and loaded on an Illumina HiSeq/Novaseq instrument according to manufacturer's instructions (Illumina, San Diego, CA, USA) or a MGI2000 instrument (MGI, Shenzhen, China). Spectral features (m/z, retention time) corresponding to identified and uncharacterized metabolites were integrated and aligned using apLCMS/xMSanalyzer software.
Data analysis	For metagenome profiling, Cutadapt (v1.9.1, http://cutadapt.readthedocs.io/en/stable/), Uparse software (v7.0.1001, http://www.drive5.com/uparse/), Mothur method and the SSUrRNA, database of SILVA132 (http://www.arb-silva.de/), QIIME software (v1.9.1), R software (v2.15.3) with the WGCNA, stats, and ggplot2 software packages, Graphviz-2.38.0, LEfSe, DIAMOND software were used. For metabolomics analysis Metaboanalyst, Mummichog, Compound Discoverer 3.0 software were used. For graphic representation and statistical analysis, SPSS 16 and GraphPad Prism 8.0 were used. Figures were edited with Adobe Illustrator (2020). All detailed software and packages are described in methods section.

For manuscripts utilizing custom algorithms or software that are central to the research but not yet described in published literature, software must be made available to editors and reviewers. We strongly encourage code deposition in a community repository (e.g. GitHub). See the Nature Portfolio guidelines for submitting code & software for further information.

Data

Policy information about availability of data

All manuscripts must include a data availability statement. This statement should provide the following information, where applicable:

- Accession codes, unique identifiers, or web links for publicly available datasets
- A description of any restrictions on data availability
- For clinical datasets or third party data, please ensure that the statement adheres to our policy

All data relevant to the study are included in the article or uploaded as supplementary information. Data are available upon reasonable request.

Human research participants

Policy information about studies involving human research participants and Sex and Gender in Research.

Reporting on sex and gender	The healthy population of longevity township consists of two biological sexes: male and female
Population characteristics	A total of 247 participants from "World Longevity Township - Jiaoling, China" were recruited, including 18 centenarians (Y120 group), 19 persons aged 81-99 (Y100 group), 77 persons aged 61-80 (Y80 group), 81 persons aged 41-60 (Y60 group), 30 persons aged 21-40 (Y40 group), and 22 persons aged 0-20 (Y20 group) (Table 1). According to the multidisciplinary health assessment, the individuals were healthy with no apparent disease.
Recruitment	Based on the national civil registration system, a total of 247 participants were randomly drawn from eight towns. Participants were eligible based on the following criteria: (1) born in Jiaoling; (2) 5 years or longer continuous residence in Jiaoling backtracking from the sampling point; (3) aged 0–110 years. All enrolled participants signed an informed consent form before their physical examination and biomaterial collection.
Ethics oversight	The study was approved by the ethics committee of The First Affiliated Hospital/School of Clinical Medicine of Guangdong Pharmaceutical University. The First Affiliated Hospital/School of Clinical Medicine of Guangdong Pharmaceutical University: 2021(13).

Note that full information on the approval of the study protocol must also be provided in the manuscript.

Field-specific reporting

Please select the one below that is the best fit for your research. If you are not sure, read the appropriate sections before making your selection.

☒ Life sciences ☐ Behavioural & social sciences ☐ Ecological, evolutionary & environmental sciences

For a reference copy of the document with all sections, see nature.com/documents/nr-reporting-summary-flat.pdf

Life sciences study design

All studies must disclose on these points even when the disclosure is negative.

Sample size	For human studies, no sample size calculations were performed. Considering the scarcity of healthy centenarians in longevity township, the sample size was chosen according to the distribution of the samples. The results obtained with centenarians are consistent with animal models and previous human studies. In mice experiments, determine the minimum number of mice required for the experiment based on previous literature.
Data exclusions	Participants meeting the following additional criteria were selected for the study: (1) with fecal and blood samples; (2) without antibiotic treatment in the past month before biomaterial collections; (3) without severe diseases (end-stage cancer, renal or liver disease).
Replication	All experiments in this manuscript were performed at least twice and demonstrated the same or similar results as those published here. Human sequencing was only performed once due to the scarcity of sample and resources, but each group has dozens of biological replicates of the same age group, and the results are in concordance with prior studies.
Randomization	Based on the national civil registration system, a total of 247 participants were randomly drawn from eight towns. And randomly assigned to six groups according to different age groups. The mice experiment is also randomly assigned to groups according to the needs of the experiment.
Blinding	Sample collection, library preparation and preliminary analysis of metagenome sequencing and metabolome studies were performed by blinded technicians and computational biologists.

Reporting for specific materials, systems and methods

We require information from authors about some types of materials, experimental systems and methods used in many studies. Here, indicate whether each material, system or method listed is relevant to your study. If you are not sure if a list item applies to your research, read the appropriate section before selecting a response.

Materials & experimental systems

n/a	Involved in the study
☒	☐ Antibodies
☒	☐ Eukaryotic cell lines
☒	☐ Palaeontology and archaeology
☐	☒ Animals and other organisms
☒	☐ Clinical data
☒	☐ Dual use research of concern

Methods

n/a	Involved in the study
☒	☐ ChIP-seq
☒	☐ Flow cytometry
☒	☐ MRI-based neuroimaging

Animals and other research organisms

Policy information about studies involving animals; ARRIVE guidelines recommended for reporting animal research, and Sex and Gender in Research

Laboratory animals	Kunming mice (SPF grade, at least 6 weeks old) were obtained from the Southern Medical University (Guangdong, China) and housed under a 12-h light/dark cycle in the gnotobiotic facilities. All mice were fed with sterile food and water ad libitum.
Wild animals	The study did not involve wild animals
Reporting on sex	Referring to previous literature of similar experiments, only male mice were selected for our mouse experiments.
Field-collected samples	The study did not involve field-collected samples
Ethics oversight	Mouse experimental procedures complied with all relevant ethical regulations and were conducted according to protocol with animal experiment ethics approval number: GT-IACUC201909026 approved by the Institute of Microbiology, Guangdong Academy of Sciences.

Note that full information on the approval of the study protocol must also be provided in the manuscript.
